# Supplementary material for: Actomyosin-mediated apical constriction promotes physiological germ cell death in C. elegans
Source: PLoS Biol. 2024 Aug 23;22(8):e3002775. doi: 10.1371/journal.pbio.3002775 (PMC11376560; doi:10.1371/journal.pbio.3002775)
Supplement: S1 Fig — (PDF) [file pbio.3002775.s001.pdf]

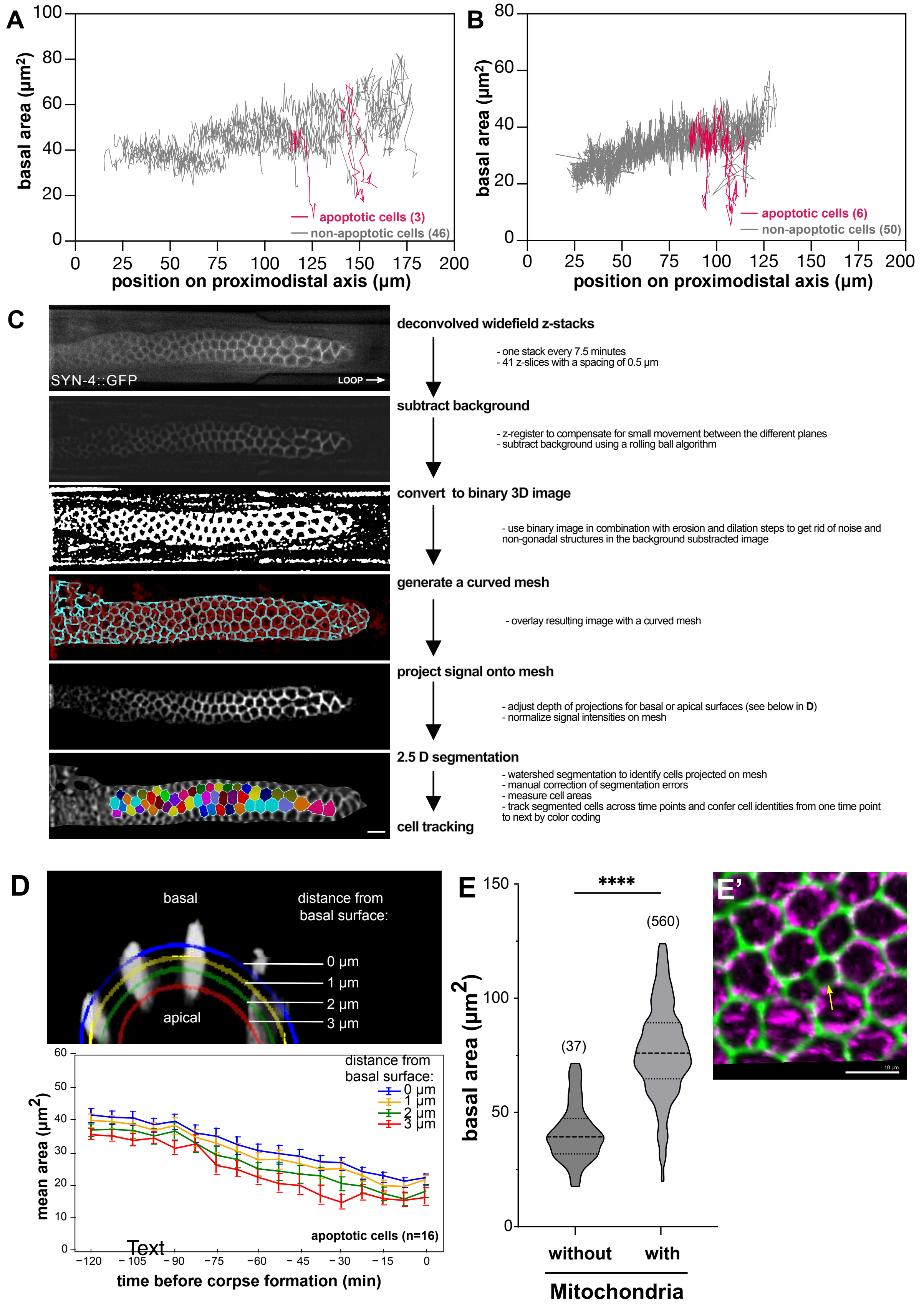

S1 Fig. related to Fig. 1

(**A, B**) Results of cell tracking experiments in two additional wild-type animals as described in **Fig. 1F**. (**C**) MorphographX workflow to track germ cells as described in the **extended methods section**. Scale bar is 10 µm. (**D**) Projections of increasing depths were generated to measure the basal, middle, and apical areas in MorphographX with the resulting plot showing the decrease in mean area ±SEM of apoptotic cell size before corpse formation in projections of increasing depths. (**E**) Violin plot showing the quantification of germ cell size in static images of the distal gonad arms. Germ cells were classified by the presence or absence of the Mito::GFP signal. The numbers in brackets indicate the numbers of cells analyzed with MorphographX in a total of 35 animals. (**E'**) Shows an example of a 2.5D projection with the membrane marker in green and the Mito::GFP signal in magenta. The yellow arrow points to a cell without mitochondria. See **S1 Data** for the raw data and statistics. The scale bar is 10 µm.
