# Supplementary material for: Actomyosin-mediated apical constriction promotes physiological germ cell death in C. elegans
Source: PLoS Biol. 2024 Aug 23;22(8):e3002775. doi: 10.1371/journal.pbio.3002775 (PMC11376560; doi:10.1371/journal.pbio.3002775)
Supplement: S2 Fig — (PDF) [file pbio.3002775.s002.pdf]

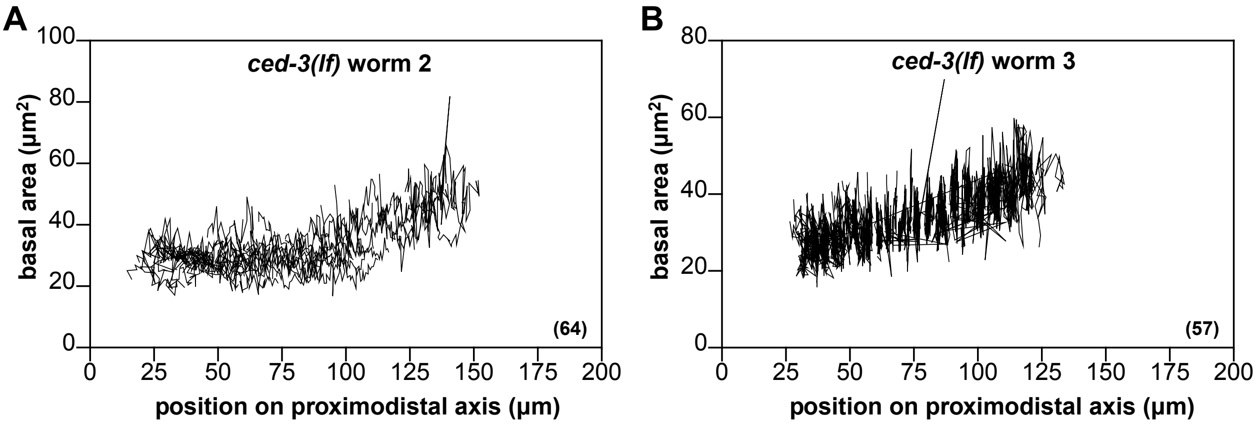

S2 Fig. related to Fig. 2
(**A, B**) Results of germ cell tracking experiments in two additional *ced-3(lf)* animals, as described in **Fig. 2A**. See **S1 Data** for the raw data.
