# Supplementary material for: Actomyosin-mediated apical constriction promotes physiological germ cell death in C. elegans
Source: PLoS Biol. 2024 Aug 23;22(8):e3002775. doi: 10.1371/journal.pbio.3002775 (PMC11376560; doi:10.1371/journal.pbio.3002775)
Supplement: S3 Fig — (PDF) [file pbio.3002775.s003.pdf]

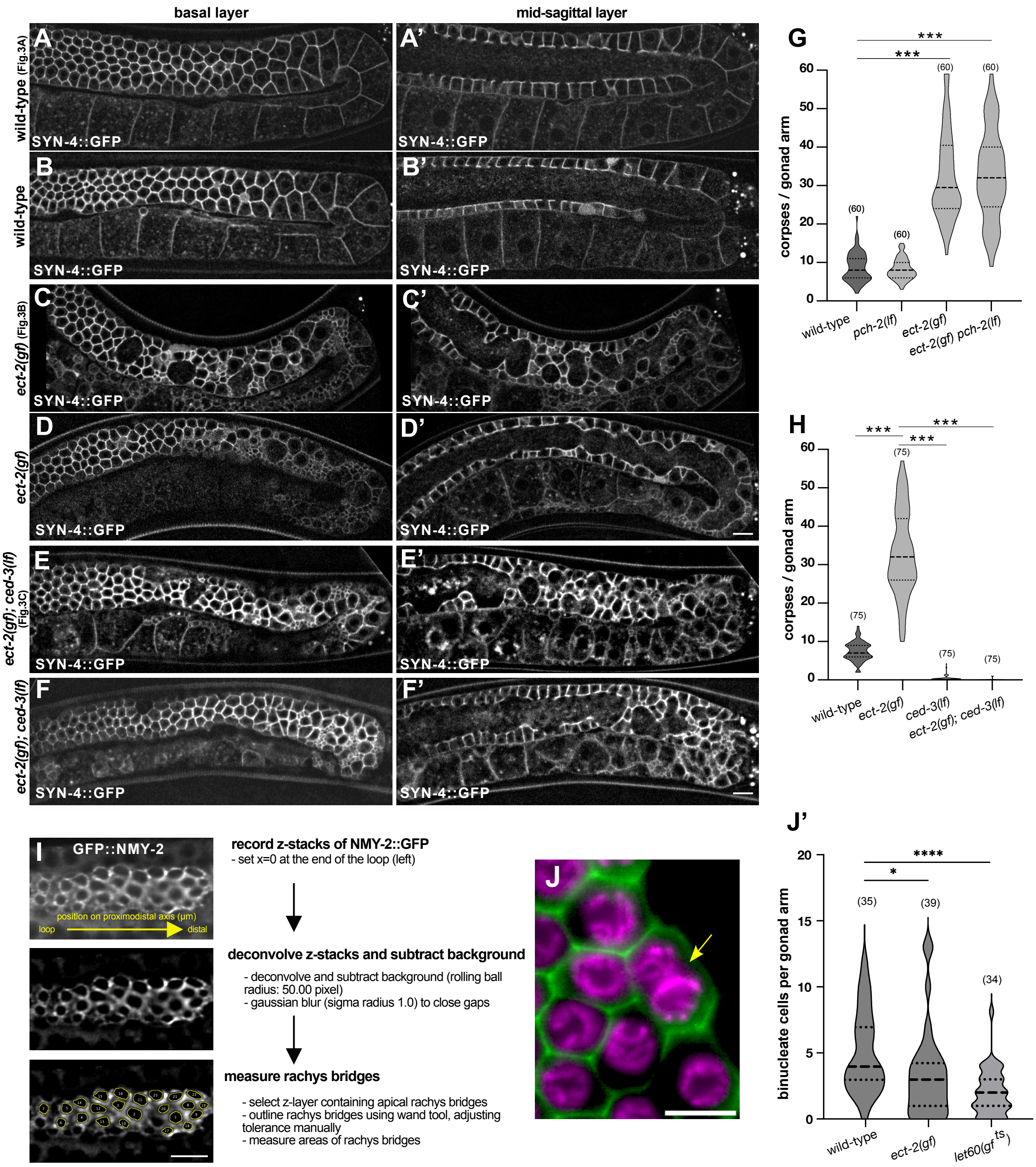

S3 Fig. related to Fig. 3

(**A-F'**) Full views of the SYN-4::GFP membrane marker in the gonad arms of the animals that are shown in **Fig. 3A-C**. For each genotype, one additional example is shown. Panels **A-F** show basal and panels **A'-F'** mid-sagittal sections. The scale bar is 10 µm. (**G, H**) Violin plots showing the number of CED-1::GFP positive apoptotic cells per gonad arm in one-day-old adults of the indicated genotypes. (**I**) Workflow used to measure the rachis bridge area in **Fig. 3G, Fig.5C, F & S5E**, as described in the **extended methods section**. (**J**) Example of a gonad dissected from an *ect-2(gf)* animal carrying the SYN-4::GFP membrane marker (green) and stained with DAPI (magenta), as described in the **extended methods**. The yellow arrow indicates a binucleate germ cell surrounded by mononucleate cells. (**J'**) Violin plot showing the number of binucleate cells per gonad arm in one-day-old adults of the indicated genotypes. Statistical analysis was done as described in the legend to **Fig. 3**. See **S1 Data** for the raw data and statistics. The scale bar is 10 µm.
