# Supplementary material for: Actomyosin-mediated apical constriction promotes physiological germ cell death in C. elegans
Source: PLoS Biol. 2024 Aug 23;22(8):e3002775. doi: 10.1371/journal.pbio.3002775 (PMC11376560; doi:10.1371/journal.pbio.3002775)
Supplement: S4 Fig — (PDF) [file pbio.3002775.s004.pdf]

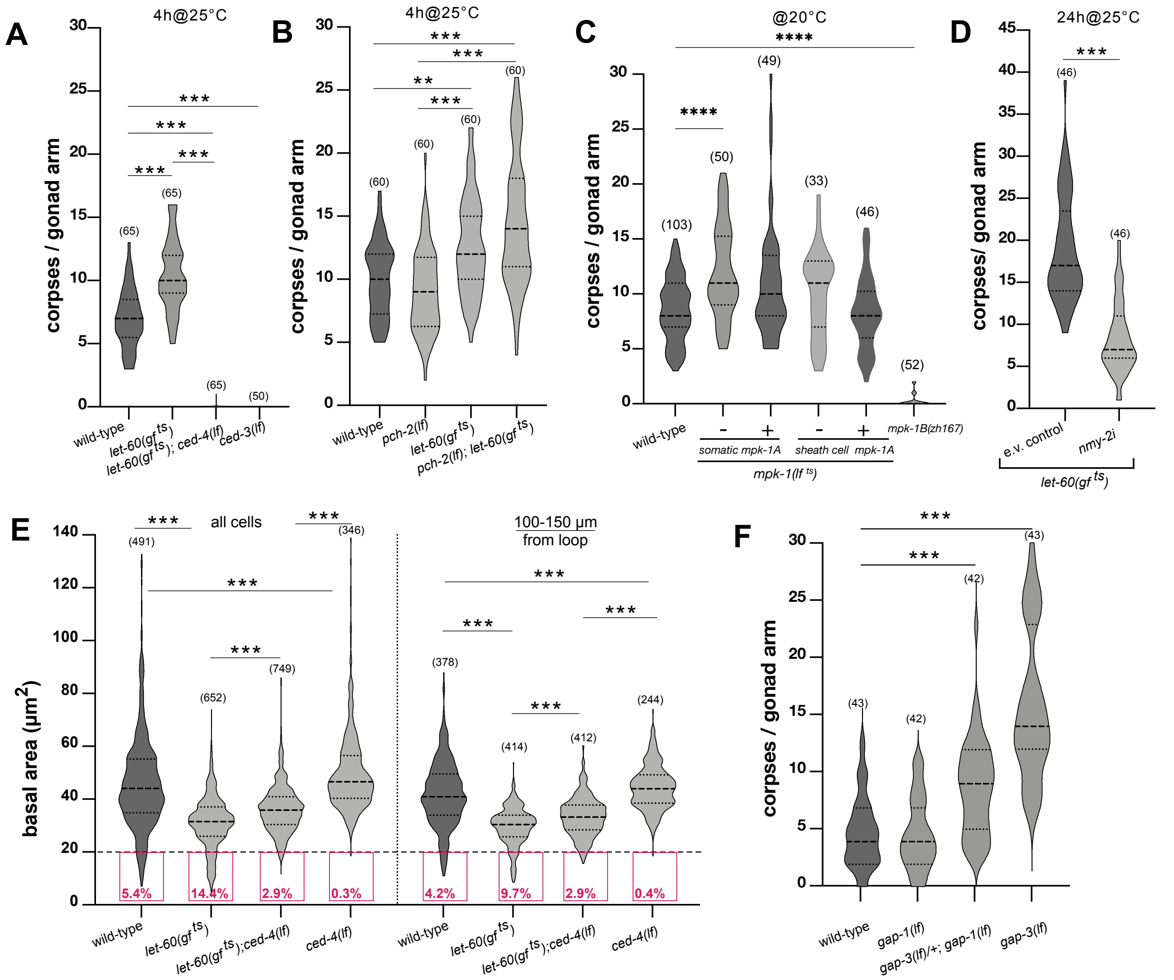

S4 Fig. related to Fig.4

(**A**) Violin plot showing the number of CED-1::GFP positive apoptotic germ cells per gonad arm in one-day-old adults of the indicated genotypes after a 4-hour up-shift to the restrictive temperature (4h@25°C). (**B**) Violin plot showing the number of CED-1::GFP positive apoptotic germ cells of the indicated genotypes grown at 20°C for 68 hours and incubated for 4 hours at the restrictive temperature of 25°C. (**C**) Violin plot showing the number of CED-1::GFP positive apoptotic germ cells per gonad arm in one-day-old adults of the indicated genotypes continuously grown at 20°C (baseline control for **Fig. 4B**, right). (**D**) Violin plot showing the number of CED-1::GFP positive apoptotic germ cells per gonad arm in one-day-old adults after *nmy-2* RNAi treatment in the *let-60(gf* ^{ts}*)* background from the L4 stage with a simultaneous 24-hour up-shift to 25°C (24h@25°C). (**E**) Violin plot for the basal cell areas in the 0 – 150 µm and 100 – 150 µm regions, besides the 50-100 µm region shown in **Fig. 4D**. (F) Violin plot showing the number of CED-1::GFP positive apoptotic germ cells per gonad arm in one-day-old adults of the indicated genotypes. *gap-3(ga139); gap-1(ga133)* homozygous double mutants had a severely disrupted gonad morphology and were therefore not scored. Statistical analysis was done as described in the legend to **Fig. 4**. See **S1 Data** for the raw data and statistics. Scale bars are 10 µm.
