# Supplementary material for: Actomyosin-mediated apical constriction promotes physiological germ cell death in C. elegans
Source: PLoS Biol. 2024 Aug 23;22(8):e3002775. doi: 10.1371/journal.pbio.3002775 (PMC11376560; doi:10.1371/journal.pbio.3002775)
Supplement: S5 Fig — (PDF) [file pbio.3002775.s005.pdf]

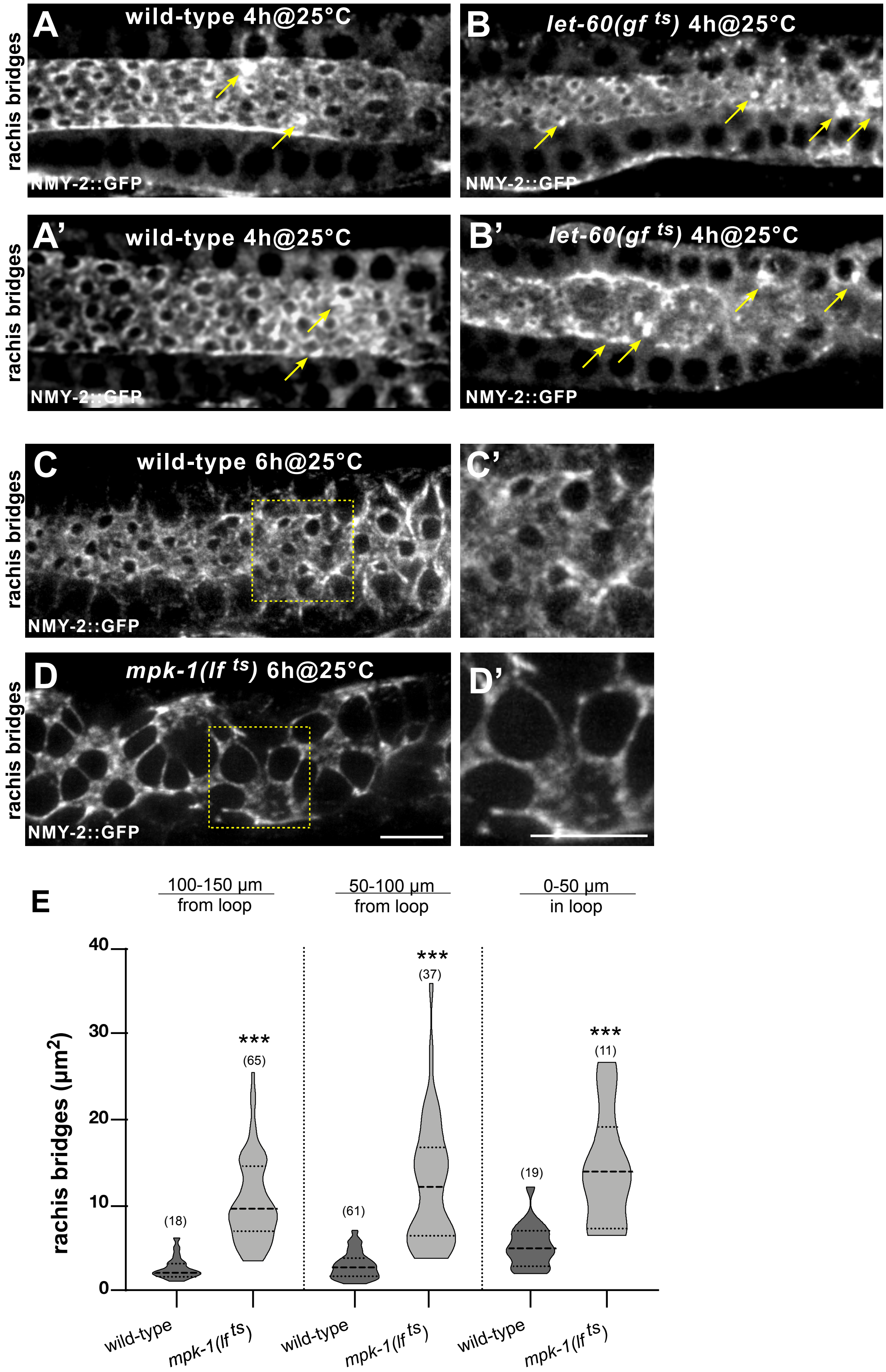

S5 Fig. related to Fig. 5

(**A-B'**) Additional examples of the rachis bridges outlined by NMY-2::GFP in wild-type and *let-60(gf^{ts})* animals. Note the yellow arrows pointing to fully enclosed rachis bridges appearing as bright NMY-2::GFP spots. (**C-D**) Rachis bridges outlined by NMY-2::GFP in young wild-type and *mpk-1(lf^{ts})* adults after a 6-hour up-shift to the restrictive temperature of 25°C. The shorter, 4 hours inactivation is shown in **Fig. 5D-E**. (**E**) Violin plots showing the size of the rachis bridges in wild-type and *mpk-1(lf^{ts})* mutants in the three indicated gonad regions after a 6-hour incubation at 25°C. Three animals were analyzed for each genotype. See **S1 Data** for the raw data and statistics. Scale bars are 10 µm.
