# Supplementary material for: Actomyosin-mediated apical constriction promotes physiological germ cell death in C. elegans
Source: PLoS Biol. 2024 Aug 23;22(8):e3002775. doi: 10.1371/journal.pbio.3002775 (PMC11376560; doi:10.1371/journal.pbio.3002775)
Supplement: S6 Fig — (PDF) [file pbio.3002775.s006.pdf]

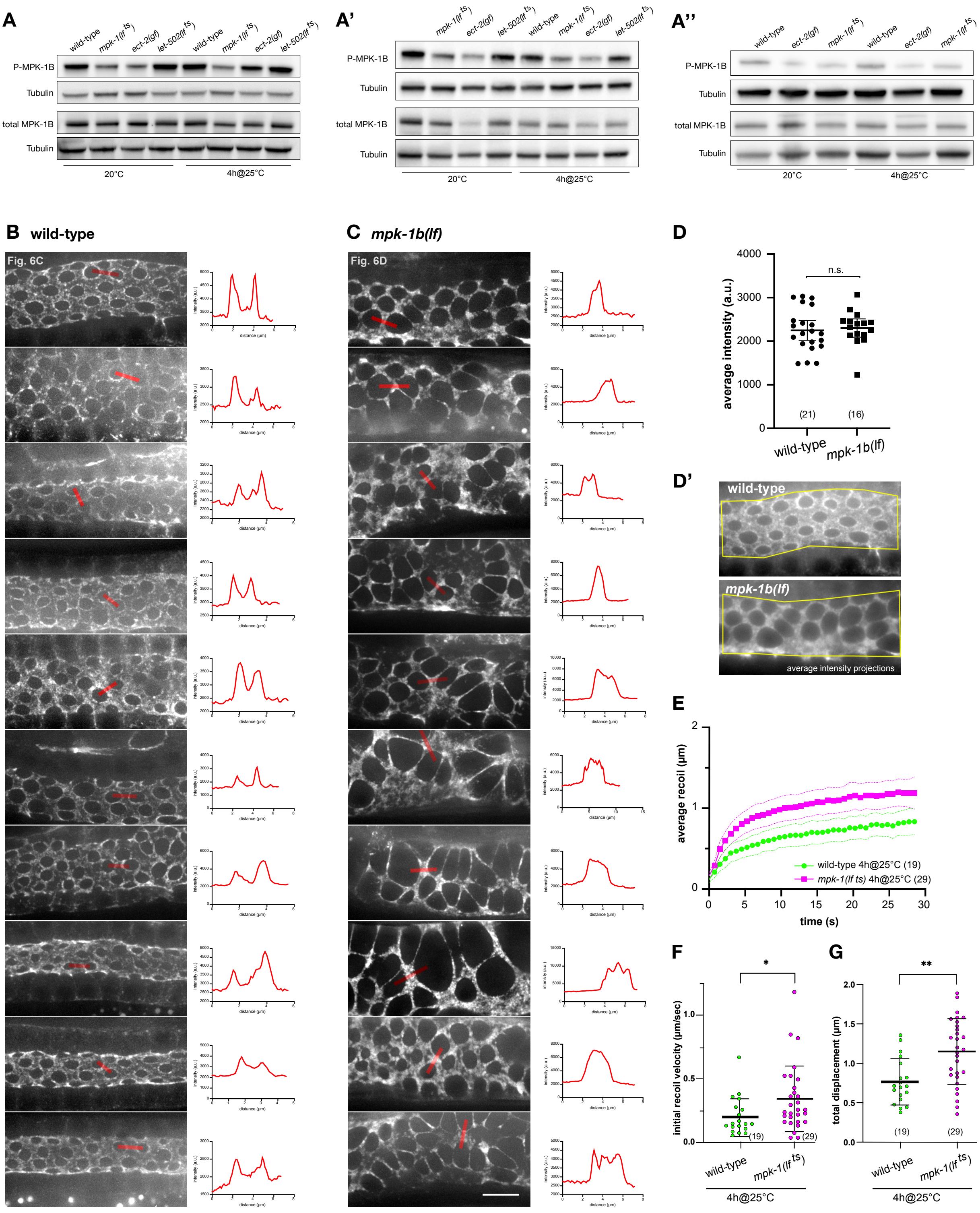

S6 Fig. related to Fig. 6

(**A**) Western blots used for the quantification of P-MPK-1B and total MPK-1B protein levels shown in **Fig. 6B**. One-day-old adults of the indicated genotypes were incubated either at 20°C for 72 hours after the L1 stage or for 4 hours at the restrictive temperature of 25°C before analysis. See **S1 raw images** for the uncropped Western blots. (**B**) Additional examples of NMY-2::GFP localization in the late pachytene region (50-100µm from the loop) of wild-type and (**C**) *mpk-1b(lf)* mutants one-day old adults (66-68 hours post-L1 arrest). Maximum intensity z-projections of flat field-corrected raw images (without deconvolution) are shown. The top panels are the same animals as in **Fig. 6C&D**. Relative intensity profiles along the regions indicated with red bars are shown to the right of each panel. Note the two peaks corresponding to the NMY-2::GFP signal at the rachis bridges in the wild-type, as opposed to the single peaks in *mpk-1b* mutants. (**D**) Quantification of NMY-2::GFP levels in wild-type and *mpk-1b(lf)* animals. Average intensity z-projections of flat field-corrected raw images were used for quantification of the mean NMY-2::GFP intensities on the apical cortex, as illustrated in (**D'**). (**E**) Mean radial displacement (recoil) plotted against time after incision, (**F**) Initial recoil velocities, and (**G**) total recoil in wild-type and *mpk-1(lf^{ts})* mutants grown for 4 hours at 25°C, calculated as described in the legend to **Fig. 6 F-H** and in materials and methods. The horizontal bars indicate the mean values, the error bars the 95% CI and the numbers in brackets the numbers of animals analyzed. See **S1 Data** for the raw data and statistics. Scale bar in (**C**) is 10 µm.
