# Supplementary figures and images for: Actomyosin-mediated apical constriction promotes physiological germ cell death in C. elegans
Source: PLoS Biol. 2024 Aug 23;22(8):e3002775. doi: 10.1371/journal.pbio.3002775 (PMC11376560; doi:10.1371/journal.pbio.3002775)

Raw images to figure S6A-A''

A

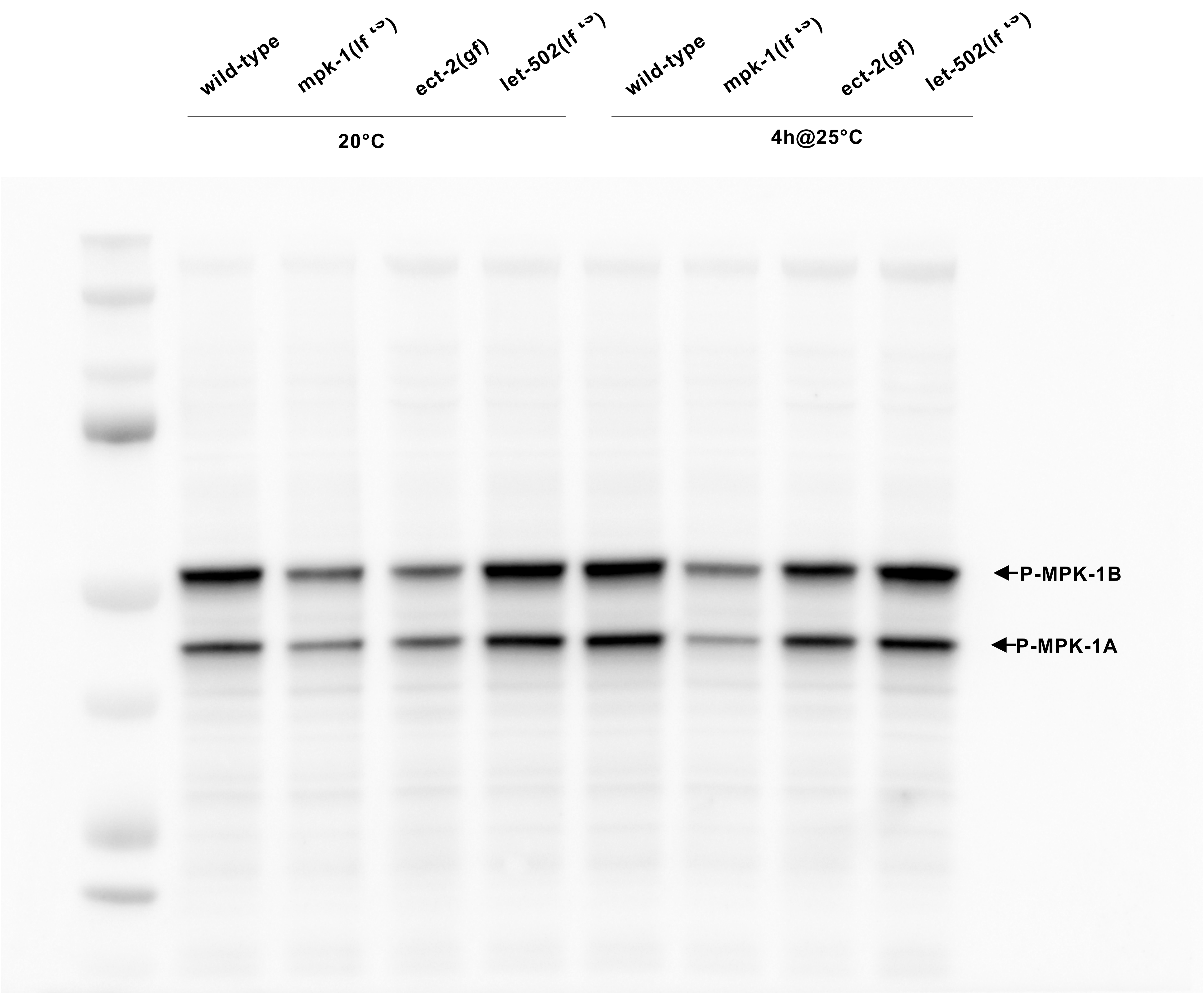

A'

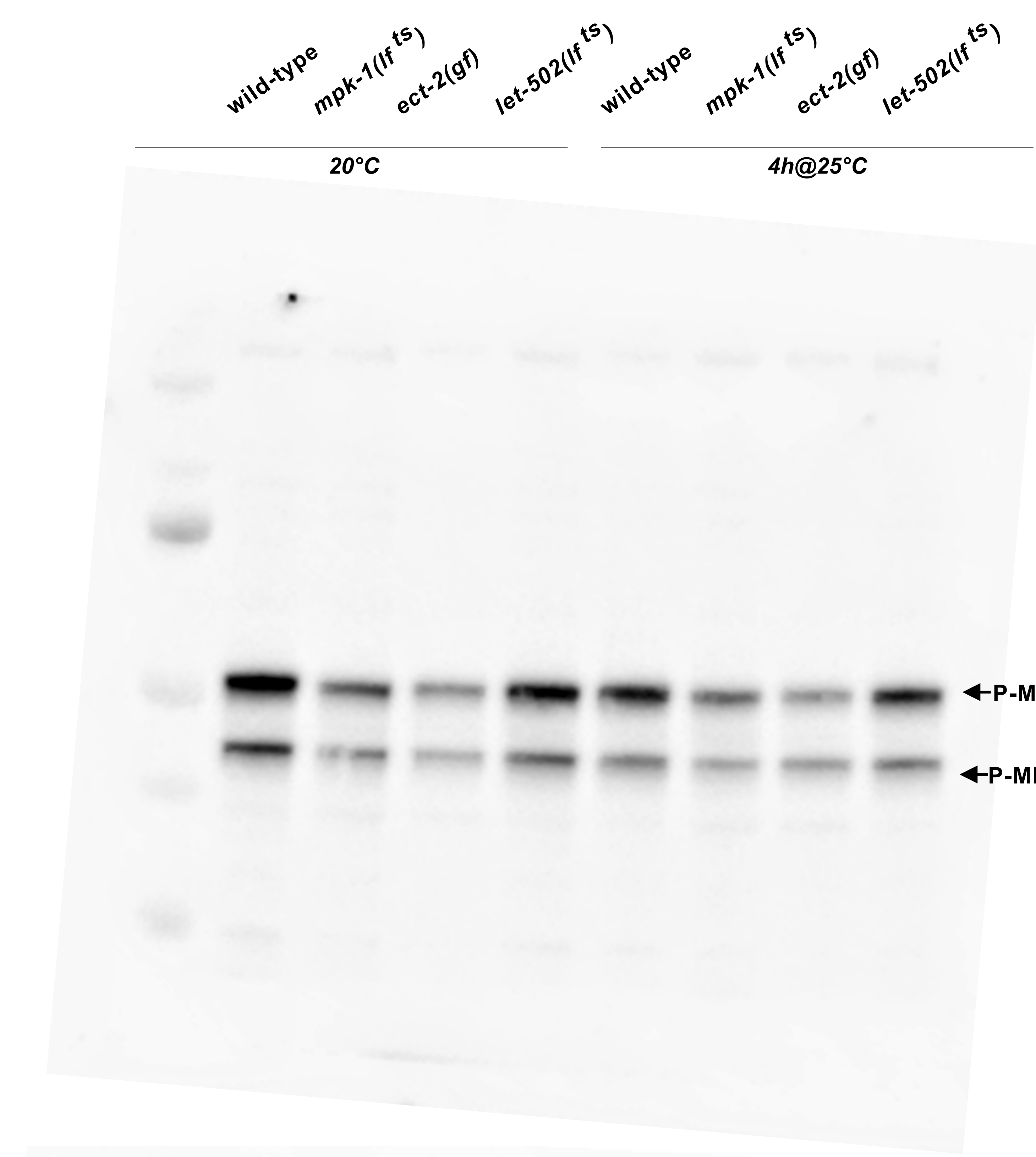

A''

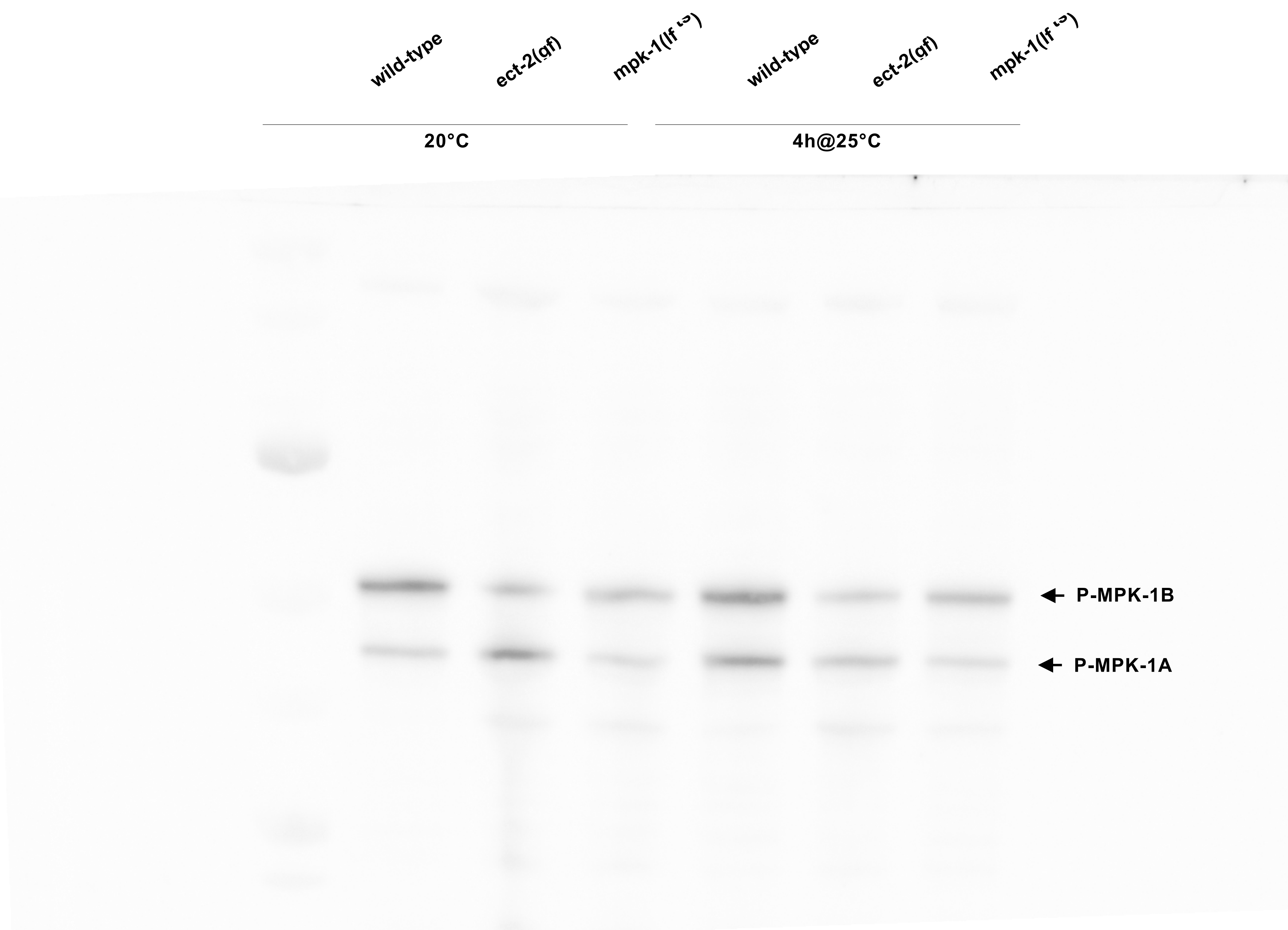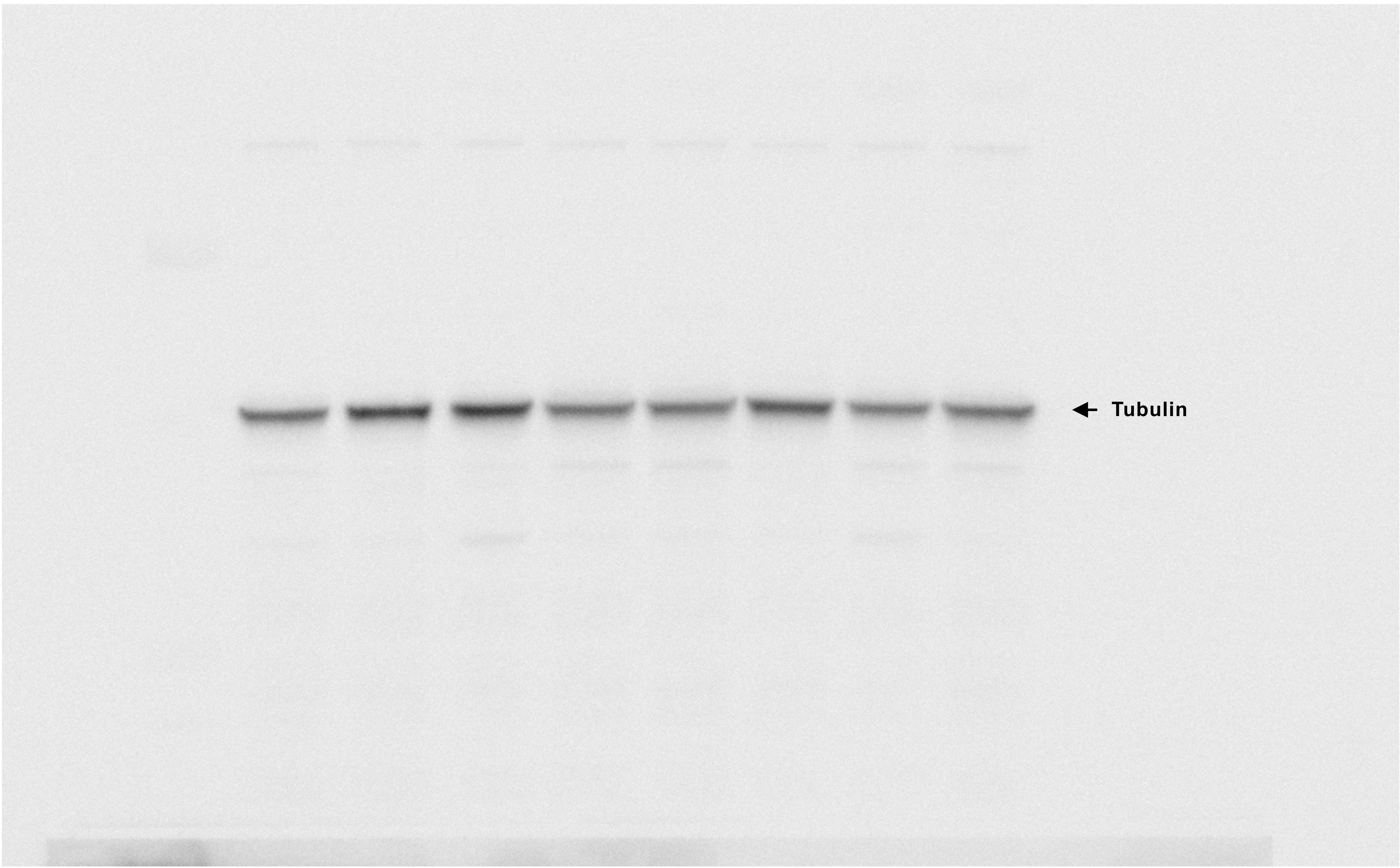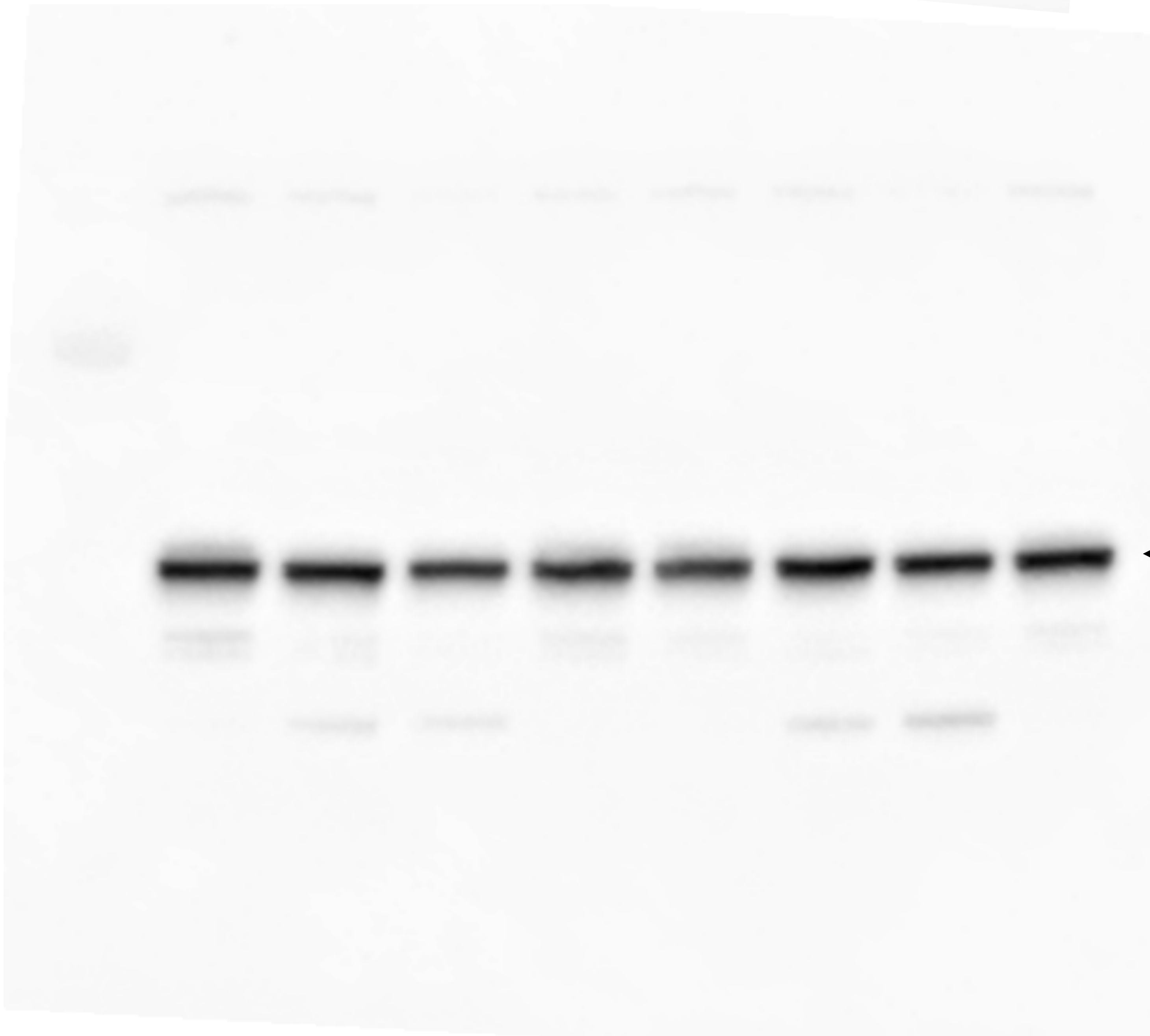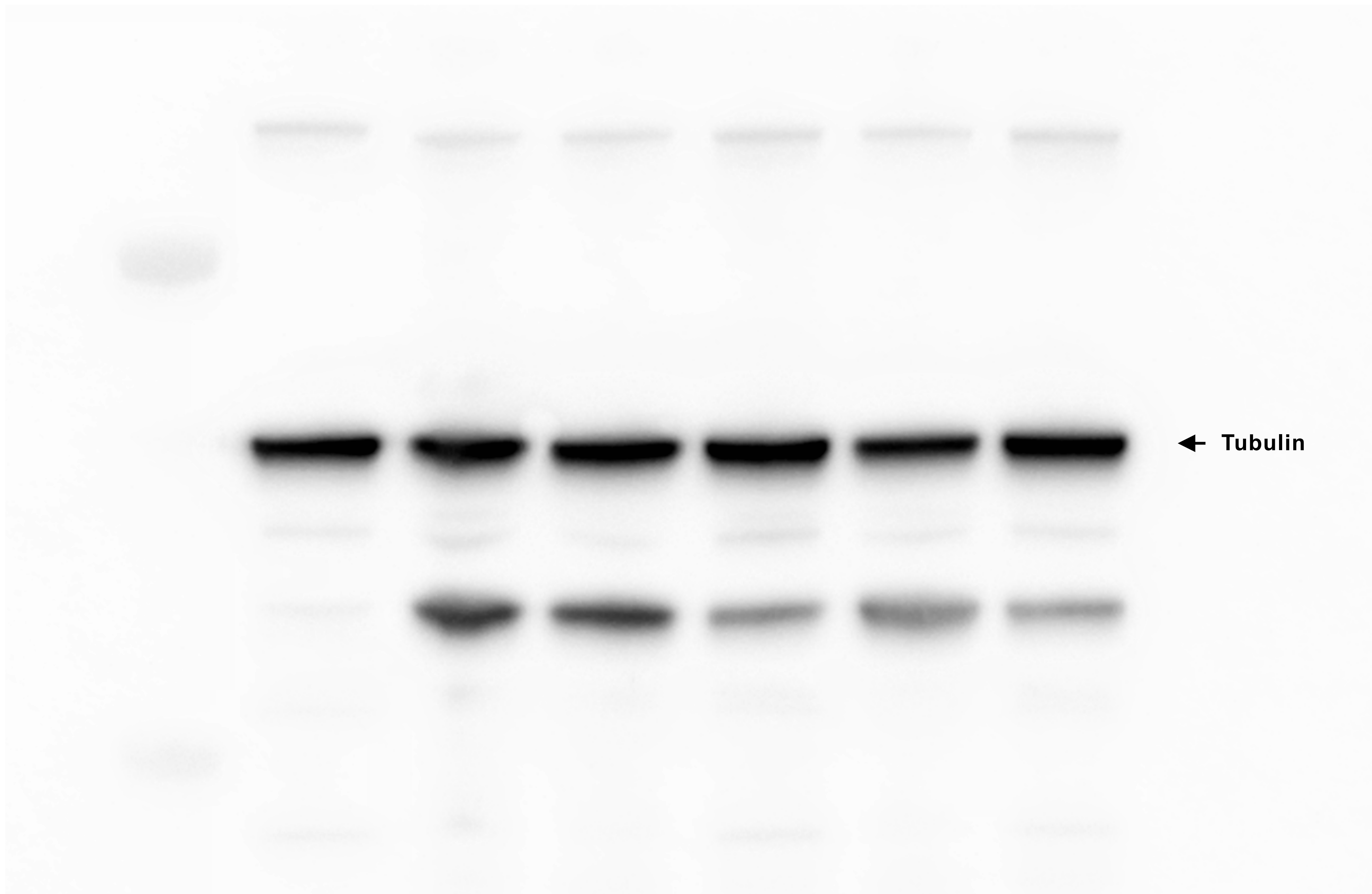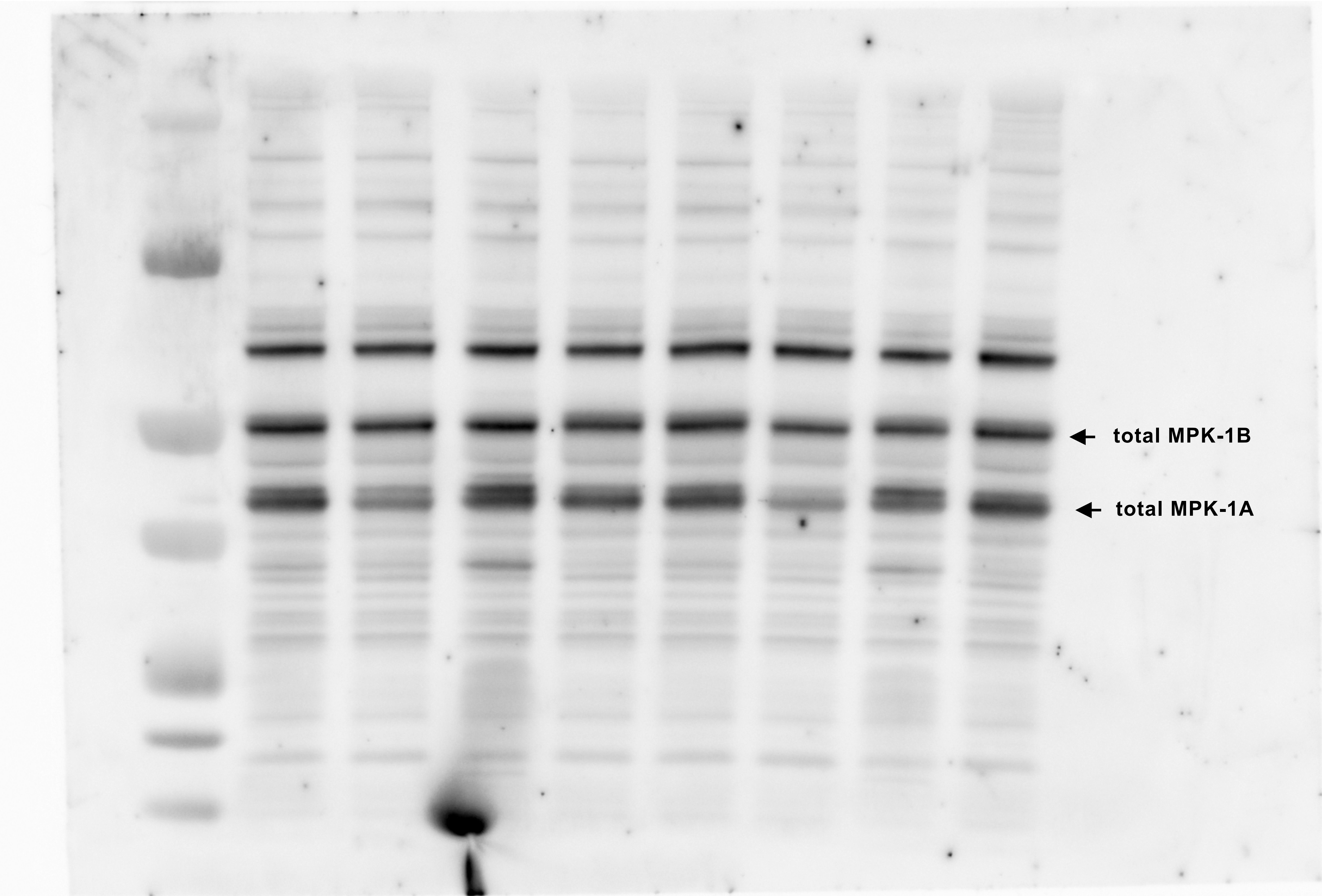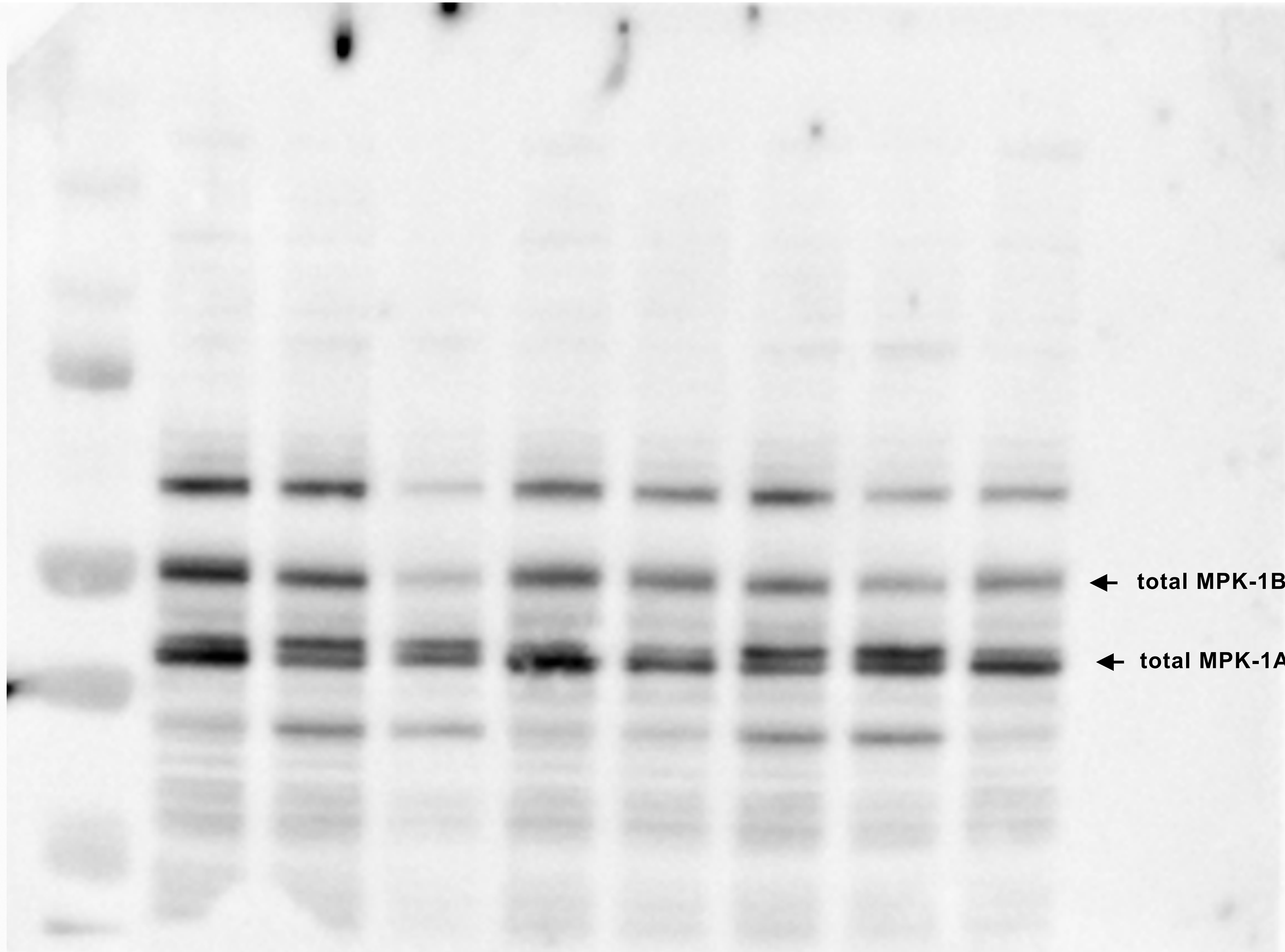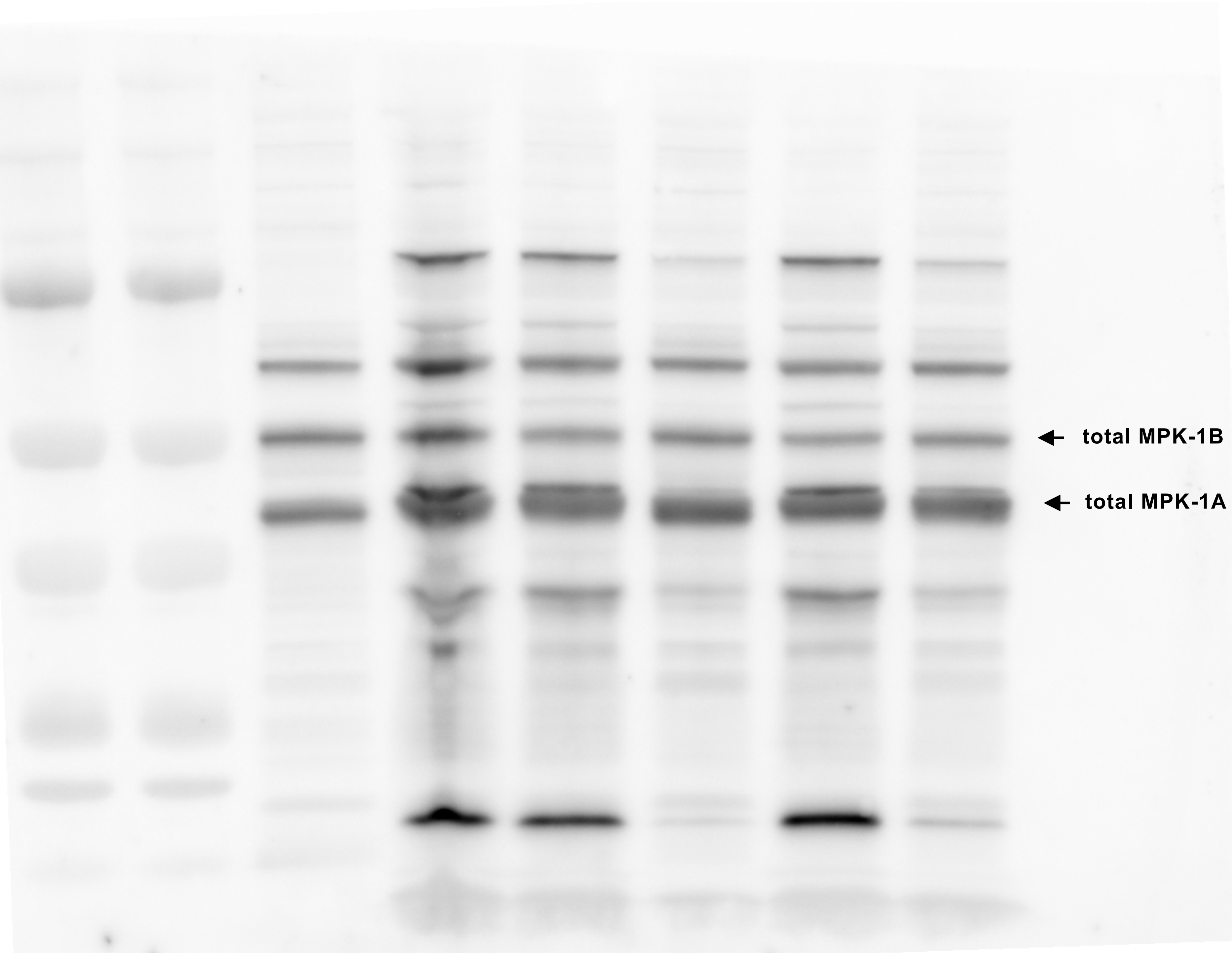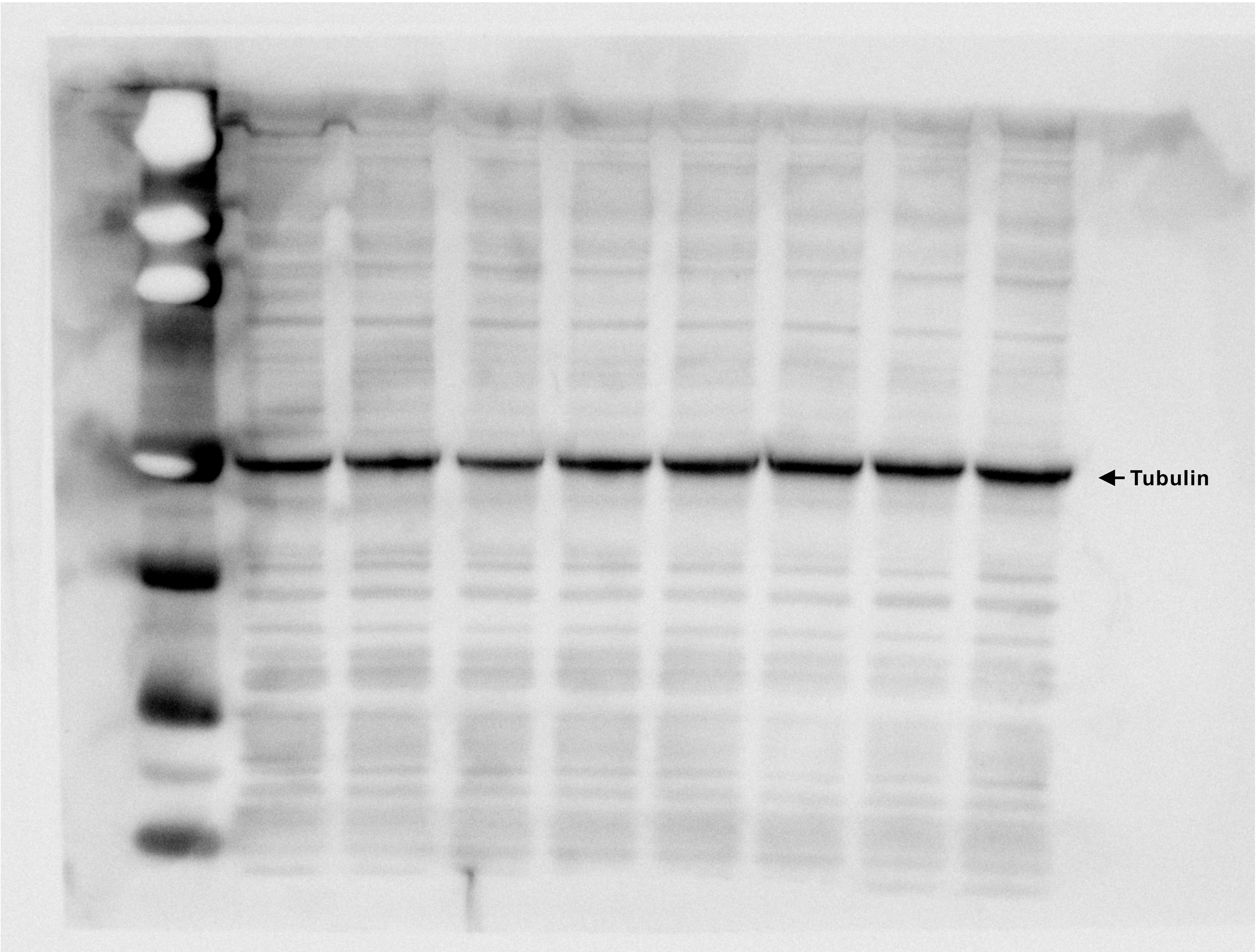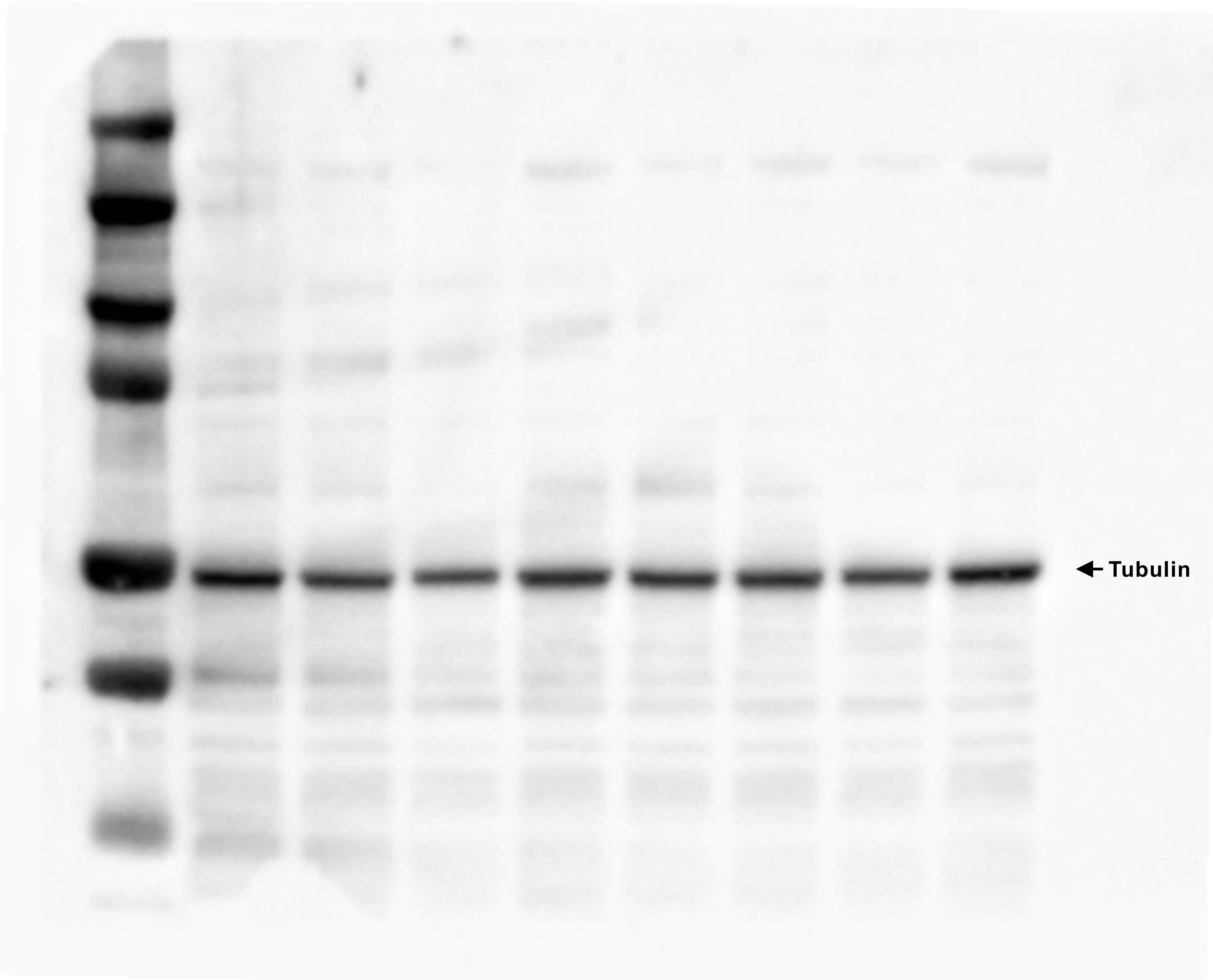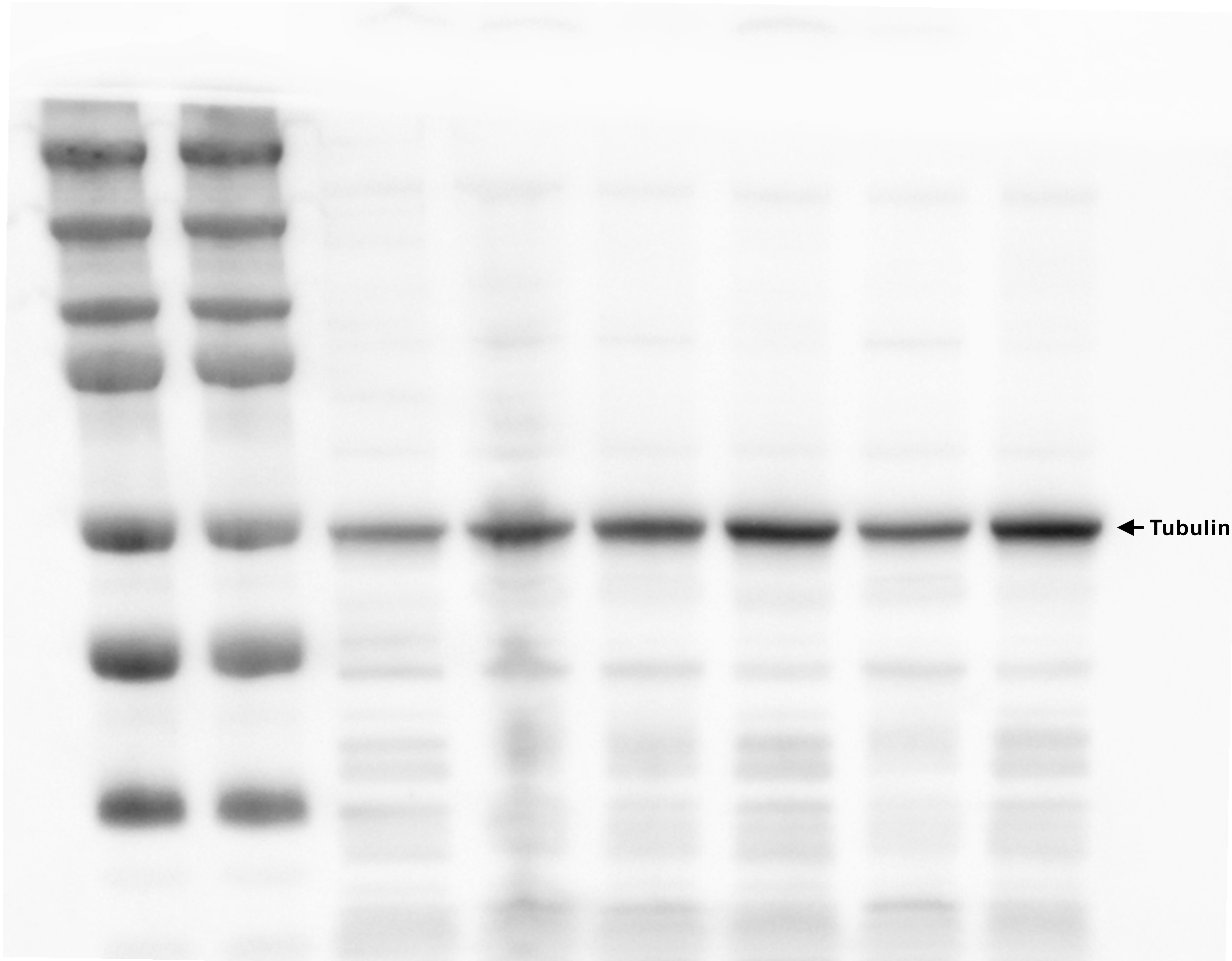

Supplement: S1 Raw images — (PDF) [file pbio.3002775.s011.pdf]
