# Supplementary material for: Actomyosin-mediated apical constriction promotes physiological germ cell death in C. elegans
Source: PLoS Biol. 2024 Aug 23;22(8):e3002775. doi: 10.1371/journal.pbio.3002775 (PMC11376560; doi:10.1371/journal.pbio.3002775)
Supplement: S1 File — (PDF) [file pbio.3002775.s015.pdf]

## Extended methods and scripts used

### Image processing

Images were processed using the Huygens Deconvolution platform (SVI, Center for Microscopy and Image Analysis, University of Zürich) or using the YacuDecu implementation of CUDA-based Richardson Lucy deconvolution in Matlab to remove background signals.

### Germ cell size measurements and tracking

*Generation of curved meshes and cell segmentation*

Pre-processing of deconvolved image stacks of the SYN-4::GFP membrane marker at each time point was done using Fiji software [70], with z-registration to compensate for small movements of the worm in the microfluidic device and additional background subtraction (see attached Fiji scripts). Cells were tracked over time using a custom-made Python script attached below with the MorphoGraphX software package [33], which enables the analysis of cell shapes on curved surfaces as illustrated in **suppl. Figure S1C**. As a first step, regions of the distal gonad arms with pachytene stage germ cells were selected and processed using a combination of dilation and erosion (the highest or lowest value of a pixel and a defined neighborhood is taken, respectively). In short, the selection was based on the assumption that germline membranes form a somewhat regular polygonal pattern. When dilating this image with a bit more than half a cell diameter, the whole gonad fills up and the opposite erosion does not reverse the image to its original, but leaves the gonad filled. Other patterns were selected against, also using the fact that the position of the worm is always very similar when using the microfluidic devices. After gonad selection, a mesh was generated based on a threshold-like mechanism that scans the image from the top to the bottom. After smoothening the mesh, its quality was checked visually and any artifacts were repaired by manual local smoothening. Such artifacts occurred in roughly 10% of the time points and were caused for example by signals that did not arise from germline cells or by small regions in the germline with relatively low signal intensity. The z-registered and background-subtracted 3D image was then projected onto the curved mesh enabling the analysis of the basal surface. Cells were segmented using a watershed algorithm for curved surfaces. The resulting segmentation of cells was checked and, if necessary, manually corrected. Areas at different distances from the basal surface (e.g. the apical surface) were obtained as follows. Using MorphoGraphX, a new mesh was created at 1, 2, or 3 μm absolute distance from the basal mesh on the apical side (**suppl. Figure S1D**). The

signal of the registered and background-subtracted image was then projected onto this new mesh and cells were again segmented using a watershed algorithm.

*Germ cell tracking on curved meshes*

The gonad arms move considerably in the microfluidic device even when the animal is largely immobile, this is necessary to allow egg laying and to maintain normal gonad physiology [12]. Therefore, cell tracking was performed manually by manually overlaying meshes of two subsequent time points and conferring cell identities from one mesh to another. In this way, it was possible to compare the patterns globally and reliably identify cells across the time points.

*Measuring cell sizes*

Basal areas of the segmented cells were obtained using MorphoGraphX, which directly measures the mesh area occupied by each cell. Basal cell areas from single images were obtained using the same scripts but with different threshold values. The segmentation of the apoptotic cells was checked manually and improved if necessary. Some cells could not be analyzed at 3 µm (or rarely at 2 µm) distance from the basal surface at some time points due to variations in cell height. In these cases, the cells were excluded from the analysis at the corresponding depths and time points. Since the membrane signal decreases most apically, the number of manual improvements increased with increasing depth. Cell areas were obtained directly from the respective meshes.

*Tracking of apical rachis bridges*

To measure the rachis bridge area labeled with NMY-2::GFP in the time-lapse recordings shown in **Fig. 1H**, the center position of each apoptotic cell was obtained from the MorphographX tracking data and used to create a cropped .tiff-file with the apoptotic cell positioned in the image center, e.g. in **Fig. 1C**, allowing us to track cells over time. The rachis bridge opening and apical cell shape of the apoptotic cell and two neighboring cells, one at the distal and one at the proximal side, were outlined manually using Fiji software [70]. For each cell, a single z-plane was selected, which was used to measure the rachis bridge and the apical membrane area. Since the rachis bridge opening is only visible in very few planes and the gonads are not completely straight, the area of a part of the neighboring cells was sometimes determined in different z-sections. To minimize systematic errors, due to the fact that the apical

surface is curved, apoptotic cells positioned in the middle of the gonads were selected for analysis as their apical surface is relatively flat.

*Tracking of mitochondria content*

Apoptotic cells were manually tracked from the appearance of an apoptotic corpse backward in time. Cell area and mitochondria content were manually quantified in single z-sections with the built-in FIJI measurement tools, using the membrane marker and Mito::GFP marker respectively. In non-apoptotic cells, the Mito::GFP marker is visible throughout the entire time course as highly granular structure. Loss of mitochondria in apoptotic cells results in loss of granularity, and therefore mitochondria content was approximated as Mito::GFP signal granularity that was quantified as signal variation (standard deviation) inside the perimeter of the pre-apoptotic cells. For each time point and apoptotic cells, these values were normalized to the signal variation in two non-apoptotic neighbors.

*Registration of cell positions*

Variation in the positions of cells along the length of the gonad was decreased by a registration-like process: two cells far apart and located in the regular part of the tissue were selected and tracked throughout the movie. Cell positions were translated such that the position of the first cell changed linearly over time. This was achieved using a linear fit between the time and x-position of this first cell. Subsequently, the x-axis was scaled such that the distance between the first and second cell increased linearly over time and the position of the first cell was not changed. This scaling was based on a linear fit between time and distance between the first and second cell.

*Measurement of rachis bridge areas in confocal image stacks*

The areas of the rachis bridge were measured using Fiji software [70], as illustrated in **suppl. Fig. S3I**. Deconvolved or unprocessed confocal z-stacks were rotated and cropped to set the x-position (x=0) to the end of the gonad loop. The background was subtracted (rolling ball radius 50) and a Gaussian Blur (sigma r=1) was applied. The wand tool was used to manually select openings (black area) of the rachis bridges and their respective areas were measured with the Fiji built-in measurement function. If necessary, the tolerance of the wand tool was adjusted when selecting the openings. Since the rachis bridge of a cell is only visible in a few z-stacks, different z-stacks were selected for different cells.

*Detection of binucleate germ cells*

The gonads of one-day-old adult wild-type, *ect-2(zh8)* and *let-60(ga89)* animals carrying the SYN-4::GFP membrane marker were dissected, fixed with 4% formaldehyde and stained for 5 minutes with 1 µg/ml DAPI (4',6'Diamindino-2-phenylindole, Sigma) to visualize the nuclei, as described [32]. *let-60(ga89)* animals were grown for 4 hrs. at 25°C before staining. Binucleate germ cells were identified in deconvolved z-stacks covering the entire distal gonad arms recorded at 0.5µm z-spacing with 40x magnification as illustrated in **suppl. Fig. S3J**.

## Scripts used for image processing

**script in Fiji used to crop and rotate single stacks (mgx preprocess.ijm, used only for single stacks analysis)**

```
{
file=fileList[f];
if (!File.isDirectory(dir+file));
{
run("Bio-Formats Importer", "open="+dir+file+" windowless");
title=getTitle();
slices=nSlices;
run("Properties...", "unit=um pixel_width=0.2 pixel_height=0.2 voxel_depth=0.66");
run("Subtract Background...", "rolling=50 stack");
//run("Remove Outliers...", "radius=3 threshold=10 which=Bright stack");
run("Gaussian Blur...", "sigma=1 stack");
run("Subtract Background...", "rolling=50 stack");
setSlice(round(slices/2));
run("Enhance Contrast", "saturated=0.35");
setTool("line");
waitForUser("Draw a distal to proximal line.");
getLine(x1, y1, x2, y2, lineWidth);
lineAngle=atan2((y2-y1),(x2-x1));
rotateAngle=(-lineAngle+PI)/PI*180;
run("Rotate... ", "angle="+rotateAngle+" grid=0 interpolation=Bicubic enlarge stack");
setTool("polygon");
waitForUser("Crop the gonad");
run("Crop");
run("Clear Outside", "stack");
waitForUser("select the bottom layer");
bottom=getSliceNumber()-1;
waitForUser("select the top layer");
top=getSliceNumber()+1;
run("Slice Remover", "first="+top+" last="+slices+" increment=1");
run("Slice Remover", "first=1 last="+bottom+" increment=1");
setSlice(round(nSlices/2));
run("Enhance Contrast", "saturated=0.35");
path=resultDir+title+"_cropped.tif";
saveAs("Tiff", path);
close();
}
}
```

**Script in Fijii to remove background from single stacks and germ cell tracking stacks (pipe_a_fiji_background.ijm)**

```
showMessage("Choose the first file (must end with '1.tif') to subtract the background from. Other files must have same naming convention.")
open("")
image_name = getInfo("image.filename")
core_input_name = split(image_name, "(1.tif)")
core_input_name = core_input_name[0]
input_directory = getInfo("image.directory")
close();
showMessage("Choose the last file (must start with "+core_input_name+") to subtract the background from.")
open("")
image_name = getInfo("image.filename")
last_number = split(image_name, "(.tif)")
last_number = split(last_number[0], "("+core_input_name+")")
last_number = last_number[1];
last_number = parseInt(last_number);
close();
target = getDirectory("Choose directory to save background-subtracted images to (eg meshes_etc)");

for (i=1; i<last_number+1; i++) {
		open(input_directory+"/"+core_input_name+i+".tif");
		run("Gaussian Blur...", "sigma=2 stack");
		run("Subtract Background...", "rolling=20 stack"); //changed from 10 to 20
		run("Remove Outliers...", "radius=2 threshold=50 which=Bright stack");
		run("StackReg", "transformation=[Rigid Body]");
		saveAs("Tiff", target+"/a_no_background_"+i+".tif");
		close();
		}
```

**Script in Python to generate 2.5D projections and segmented cells in MorphographX (pipe_b_morphographx_segment)**

```
"""Second step of segmentation and tracking of germline movies (with MorphoGraphX):
- It performs dilation and erosion steps to get rid of (most) signals that do not arise from germ cells before the turn of the gonad.
- It creates a curved mesh (2.5D) through basal membranes
- It projects the image signal on this mesh and segments the projections by watershedding.
Prerequisites: folder with .tif files generated by pipe_a_fiji_background.ijm
Before running the code from MorphoGraphX, adjust the two paths in the first lines below
"""

srcDir= # folder containing the no_background files
targDir= # folder where to save the files
for i in range(1,2):
   Stack.Open(srcDir+'a_no_background_'+str(i)+'.tif', 'Main', '0')
   Global.SetCurrentStack('Main', '0')
   Stack.Binarize('10') #This variable is not always the same: wt, ced: 10; nmy2: 80 or 200
   Stack.Save(targDir+'b_binary_'+str(i)+'.tif', 'Work', '0', '5')
   Stack.Copy_Work_to_Main_Stack()
   Global.SetCurrentStack('Main', '0')
   Stack.Change_Voxel_Size('0.21', '0.21', '0.5')#depends on resolution movie/stacks
   Stack.Reverse_Axes('No', 'No', 'Yes')
   Stack.Copy_Work_to_Main_Stack()
   Global.SetCurrentStack('Work', '0')

##Get rid of background noise
   Stack.Erode('1', '1', '1', 'No')
   Stack.Dilate('1', '1', '1', 'No')

##Get rid of horizontal lines
```

```
    Stack.Dilate('12', '0', '0', 'No')
    Stack.Erode('0', '10', '0', 'No')
    Stack.Dilate('0', '10', '0', 'No')
    Stack.Combine_Stacks('Product')

##Get rid of vertical lines and structures that are not continuous in the x direction
    Stack.Copy_Work_to_Main_Stack()
    Stack.Dilate('12', '1', '0', 'No')
    Stack.Erode('25', '10', '0', 'No')
    Stack.Dilate('25', '10', '0', 'No')
    Stack.Open(srcDir+'a_no_background_'+str(i)+'.tif', 'Main', '1')
    Global.SetCurrentStack('Main', '1')
    Stack.Change_Voxel_Size('0.21', '0.21', '0.5')#depends on resolution movie/stacks
    Stack.Autoscale_Stack()
    Stack.Apply_Transfer_Function('0', '0', '0', '1')
    Stack.Reverse_Axes('No', 'No', 'Yes')
    Stack.Copy_Work_to_Main_Stack()
    Stack.Swap_or_Copy_Stack_1_and_2('Main', '1 <-> 2')
    Global.SetCurrentStack('Work', '0')
    Stack.Combine_Stacks('Product')

##Get rid of vertical lines that span the whole gonad
    Stack.Copy_Work_to_Main_Stack()
    Stack.Erode('0', '75', '0', 'No')#gonad no longer visible
    Stack.Dilate('0', '75', '0', 'No')
    Stack.Combine_Stacks('Subtract')
    Stack.Save(targDir+'c_gonad_only_'+str(i)+'.tif', 'Work', '0', '5')

##Edge detect
    Stack.Copy_Work_to_Main_Stack()
    Stack.Edge_Detect('70.0', '2.0', '0.3', '15000')
    Stack.Cimg_Median_Blur('3')
    #Close holes
    Stack.Dilate('25', '0', '0', 'No')
    Stack.Erode('25', '0', '0', 'No')

##Generate Mesh
    Stack.Copy_Work_to_Main_Stack()
    Mesh.Marching_Cubes_Surface('3', '5000')
    Mesh.Smooth_Mesh('12')
    Mesh.Subdivide()
    Mesh.Smooth_Mesh('10')
    Mesh.Subdivide()
    Mesh.Smooth_Mesh('10')

##Projecting the original image after background subtraction on mesh
    Stack.Open(srcDir+'a_no_background_'+str(i)+'.tif', 'Main', '0')
    Global.SetCurrentStack('Main', '0')
    Stack.Change_Voxel_Size('0.21', '0.21', '0.5')
    Stack.Autoscale_Stack()
    Stack.Apply_Transfer_Function('0', '0', '0', '1')
    Stack.Reverse_Axes('No', 'No', 'Yes')
    Stack.Copy_Work_to_Main_Stack()
    Global.SetCurrentStack('Work', '0')
    Mesh.Project_Signal('No', '0', '5', '0.0', '60000.0')
    Mesh.Difference_of_Gaussians('1.0', '5.0')

##Watershed segmentation of projected signal
    Mesh.Auto_Seeding('3.0')
    Mesh.Watershed_Segmentation('20000')
```

```
   Mesh.Fix_Corners('Yes', 'Yes', '5')

##For generating snapshots to check the quality and saving the mesh
   Mesh.Normalize_Signal('10')
   Global.Snapshot(targDir+'meshview_'+str(i)+'.png', 'false', '0', '0', '1.0', '95')
   Mesh.Save(targDir+'mesh_'+str(i)+'.mgxm', 'no', '0')

##If mesh quality was not satisfactory, the mesh was improved manually and projection and subsequent steps were repeated
##In addition, segmentation was improved manually if necessary. In that case, that steps after the segmentation were repeated
```
