# Supplementary material for: Actomyosin-mediated apical constriction promotes physiological germ cell death in C. elegans
Source: PLoS Biol. 2024 Aug 23;22(8):e3002775. doi: 10.1371/journal.pbio.3002775 (PMC11376560; doi:10.1371/journal.pbio.3002775)
Supplement: Attachment — Submitted filename: point-by-point reply.pdf [file pbio.3002775.s016.pdf]

To reviewer 1

Summary: Programmed cell deaths play a vital part in the development and homeostasis of all animals. This role is particularly important in the female germ line. Although the genetic control of cell death was first described in the nematode C. elegans decades ago, the regulation of germ cell deaths in this animal is complex and remains confusing. Here the authors combine a wide array of experimental techniques to support the model that smaller germ cells are preferentially eliminated by programmed cell deaths late in the pachytene phase of meiosis I, and that their small size appears to determine cell death, rather than being an early cause of it. They dissect the regulation of germ cell size by the ECT/RHO/ROCK and RAS/MAPK pathways, and show that these genes control the constriction of the actomyosin network that determines the size of the pores connecting each germ cell to the central core of the syncytium. These results are an important advance for understanding cell death in nematodes and in germ cells, and could have much broader implications.

Recommendation: This is a beautiful, detailed study of broad interest to readers of PLoS Biology. It is based on extensize and sophisticated experiments, and the arguments unfold in a very logical and organized manner. I strongly recommend publication.

Comments:

(1) The mpk-1b phenotype is so large in figures 4B and 6A, that I wonder if the somatic effects of MPK-1a not only involve corpse clearing, but also the decision to die.

---

zh164 is a constitutive null allele while *ga111*^{ts} is reduction-of-function allele. Moreover, for *ga111* we only induced a transient, 4-hour inactivation at 25°- we think this largely explains the much stronger effect of *zh164*. Also, the gonad architecture (i.e. opening of the rachis bridges) was more strongly affected in *zh164* (**Fig 6D**) than in *ga111* animals after 4hrs @25° (**Fig. 5E**). Somatic *mpk-1a* may induce enhanced apoptosis, since corpse clearing promotes death under certain conditions, when cells are at a threshold to die (*Reddien et al. 2001)*, plus somatic *mpk-1a* is needed for germ cell proliferation (ref 53). However, all these known somatic *mpk-1a* functions cannot account for the almost complete absence of corpses in *zh164*.

It would be very helpful for the reader if the authors would extend their description of the mpk-1b mutant to a survey of all germline and somatic gonad phenotypes, so the reader could consider whether other big interactions might also being altered.

No obvious somatic phenotypes were observed in *zh164* mutants (**line 413f**). Only the germline seems to be affected with a disruption of the rachis bridges being the most obvious phenotype (**Fig. 6 C,D**), which may lead to the overall disrupted gonad architecture combined with other *mpk-1* functions (see **lines 697 & 709f**). Interestingly, gonad architecture in late L4 and very young adults (until 55 to 57 hours post L1 arrest) was largely normal in *zh164* mutants. This period corresponds to the time when the accumulation of NMY-2 at the rachis bridge in late pachytene can first be observed in the wild-type.

(2) In lines 611-612, the authors write "In the C. elegans hermaphrodite germline, RAS/MAPK
signaling plays an essential role at two stages..." However, Lee et al 2007 identified four roles for mpk-1 in the germ line.

We have changed this section and added the reference – though the role of *mpk-1* in germ cell proliferation was recently shown to be a somatic function (**line 676f**).

(3) When the authors first mention 2.5D projections, they should describe them briefly.

We used Morphographx to create projections on a curved 3D mesh around the gonads (see addition on **line 146**). In **Fig S1C** we show the detailed workflow to generate the 2.5 projections, and the python code that can be run directly in Morphographx is attached at the end of the **suppl. Information**.

Corrections

Line 40 Change "before they can enter" to "before they can complete"

corrected

Line 51 Change "1'000" to "1000"

corrected

Line 151 Change "are also labeled in black." to "are also labeled in gray."

corrected

Line 312 Change "let-60 ras(ga89ts)" to "let-60 ras(ga89ts)"

corrected, the abbreviation *let-60(gfts)* is now consistently used

Line 446 Change "mpk-1(ts lf)" to "mpk-1(lf ts)"

corrected

Line 466 Change "mpk-1(gf ts)" to "mpk-1(lf ts)"

Line 615 Change corrected "the membrane integrity," to "membrane integrity,"

corrected

To reviewer 2

Summary

Programmed cell death or apoptosis is an important cell fate in developmental biology, needed to sculpt tissues and organs. A key question is how cells adopt the apoptotic fate. Studies in C. elegans have pioneered the field. In the C. elegans soma, expression of the proapoptotic BH3-only protein EGL-1 is a major determinant of the apoptotic cell fate choice. By contrast, in the C. elegans germline in which ~60-80% of oogenic germ cells undergo apoptosis, EGL-1 is dispensable for the apoptotic cell fate choice. How female germ cells are allocated to this cell fate is an unanswered question in the field. This study from the Hajnal lab presents a new model in which the RAS/MAPK pathway functions in parallel to an ECT/RHO/ROC actin regulatory pathway upstream of CED-3 Caspase activation to allocate female germ cells to the apoptotic pathway (see their Figure 7).

Critique
I am not convinced by the data in support of the authors' model. Discrepancies with prior publications have not been adequately addressed in the manuscript. Physiological cell death in the C. elegans germline proceeds in three phases: induction, execution, and clearance. The shrinkage of apoptotic germ cells has been well documented in prior studies. This actin-dependent shrinkage of germ cells is generally thought of as being a key part of the execution phase. Importantly, the earliest reported phene of apoptotic germ cells is that they export mitochondria to the germline core cytoplasm (rachis) in a microtubule-dependent manner (Raiders SA, Eastwood MD, Bacher M, Priess JR. Binucleate germ cells in Caenorhabditis elegans are removed by physiological apoptosis. PLoS Genet. 2018 Jul 19;14(7):e1007417. doi: 10.1371/journal.pgen.1007417. PMID: 30024879; PMCID: PMC6053125.). This early mitochondrial export is then followed by the shrinkage of germ cells and their engulfment by the somatic sheath cells. In their studies, the authors have not examined the earliest stages of the apoptotic process—mitochondrial export. An alternative possibility is that the experimental perturbations are augmenting the execution phase.

1. Does germ cell shrinkage occur early or late during execution of apoptosis?

This is an important point. As stated, mitochondrial export is the earliest sign of the initiation of apoptosis. Since Raiders et al (ref 32) did shorter time lapse recordings (around 60 minutes) and did not quantify cells sizes, we have now performed long-term imaging (up to 300min) and quantified germ cell size together with mitochondria content. These new experiment **(Fig 1. L-O)** indicate that germ cell shrinkage and loss of mitochondria occur simultaneously (**line170f**). Also, the analysis of nearly 600 cells in static images confirms this conclusion **(suppl. Fig S1 E).**

While the data shown in **Figs. 1 & S1** show a clear correlation between small cell size and the onset of apoptosis, we state explicitly on **line 183f** that these data do cannot distinguish between cause and consequence.

The experiments presented in the manuscript are not sufficient to position the ECT/RHO/ROC actin regulatory pathway upstream of CED-3. It is also possible that the experimental treatments cause syncytial germ cells that are near the threshold of CED-3 caspase activation to commit to the apoptotic cell fate. In this manner, a

downstream step in the execution step might feedback on the decision. There is precedent for such a mechanism in the C. elegans soma. For example, Reddien et al. (2001) showed that engulfment, which is a step downstream of caspase activation, could promote apoptosis when ced-3 activity was limiting. (Reddien PW, Cameron S, Horvitz HR. Phagocytosis promotes programmed cell death in C. elegans. Nature. 2001 Jul 12;412(6843):198-202. doi: 10.1038/35084096. PMID: 11449278). As the authors acknowledge, their experimental perturbations of both the actin regulatory pathways and the RAS/MAPK pathway are highly pleiotropic to the germline.

---

2. Does *ect-2* act upstream or downstream of the CED pathway?

We used the canonical *ced-3(n717)* null allele in all our experiments. Also, the *ced-4* allele (*n1162*) used for the let-60(gf) double mutant is a null. Hence, residual *ced* pathway activity near the threshold activated by a positive feedback loop in *ect-2(zh8); ced-3(n717)* double mutants cannot explain our data. Thus, the small cells observed in *ect-2(gf)* (**Fig. 3**) and in *let-60(gf)* mutants (**Fig. 4**) form independently of the CED pathway (**lines 272f and 448f**). The germline-specific *ect-2* RNAi experiments indicate that activation of the ECT2/RHO/ROCK pathway in the germ cells is necessary to promote apoptosis. Hence, activation of an ECT2/RHO/ROCK feedback loop from the engulfing sheath cells by can be ruled out. On the other hand, the experiments shown in **Fig. 2** indicate that activation of the CED pathway further decreases cell size of dying cells by recruiting actomyosin components around the dying cells, which is now indicated in the model **Fig. 7** with a feedback arrow and discussed on **lines 220f and lines 642**.

It is also possible that the experimental treatments cause syncytial germ cells that are near the threshold of CED-3 caspase activation to commit to the apoptotic cell fate.

We assume this comment refers to the binucleate cells described by Raiders et al. To address this issue, we have quantified the numbers of binucleate cells in *ect-2(gf)* and *let-60(gf)* animals and found no increase compared to the wild-type (and even a decrease in *let-60(gf)* (**suppl. Fig S3J, J'**). This indicates that the enhanced apoptosis observed in *ect-2(gf)* and *let-60(gf)* germ cells is not caused by excess formation of binucleate cells (**lines 298f and 348f**)

The authors themselves mention that the role of the RAS/MAPK pathway in physiological germ cell death is "controversial" (line 307). The manuscript inadequately presents this controversy and fails to resolve the issue. It is known that strong loss-of-function mpk-1 alleles abolish germline apoptosis; however, germ cells in these mutants arrest in the mid-pachytene stage. Since apoptosis occurs mainly among more proximal pachytene cells, it might be that these germ cells have not progressed to the stage of pachytene in which they undergo apoptosis. In fact, when mpk-1(ga111ts) mutants are shifted to the non-permissive temperature, an increased number of apoptotic germ cell corpses is observed (Arur S, Ohmachi M, Nayak S, Hayes M, Miranda A, Hay A, Golden A, Schedl T. Multiple ERK substrates execute single biological processes in Caenorhabditis elegans germ-line development. Proc Natl Acad Sci U S A. 2009 Mar 24;106(12):4776-81. doi: 10.1073/pnas.0812285106. Epub 2009 Mar 5. PMID: 19264959; PMCID: PMC2660749. See their Figure S1). The authors should cite this paper and more completely investigate whether mpk-1 promotes or inhibits (or both) the apoptotic fate using mpk-1(ga111ts) mutants and other approaches.

---

3. Distinguishing the different roles of *mpk-1* in germ cell death

This is the second critical point raised, especially since the existing literature is contradictory (refs. 11, 30, 45, 48&49). We tried to represent the facts in an unbiased fashion citing all the existing literature and have now also included the Arur et al (2009) reference (ref 49), where the increase in apoptosis observed in the same *ga111ts* mutants we have been using in this manuscript was first mentioned in Suppl Fig. S1, though without quantification. The first report showing increased apoptosis in *ga111ts* mutants with a quantification is Das et al (ref 48).

We have included several new experiments that further distinguish between the roles of the somatic *mpk-1a* and the germline-specific *mpk-1b* isoforms in germ cell death. **Fig. 4B** shows germline and soma-specific RNAi experiments, which point to the pro-apoptotic role of *mpk-1b* in the germline and suggest that somatic *mpk-1a* may be required for corpse clearing by the sheath cells (i.e. reduced corpse number after germline-specific *mpk-1* RNAi and the increase in corpse number after soma-specific *mpk-1* RNAi). These tissue-specific RNAi experiments are further supported by tissue-specific rescue experiments of the same *mpk-1(ga111ts)* allele that was used in all the other references (11, 30, 45, 48 & 49) and also in our original version of the manuscript (see revised **Fig. 4C** and original **Fig. 4B**). As before, we induced a short and transient inactivation with a 4 hour up-shift to the non-permissive temperature. Unlike the constitutive *zh164* allele, this brief inactivation affects the cells that are already in late pachytene and are therefore competent to undergo apoptosis. Under these conditions (and also upon global *mpk-1* RNAi, **Fig. 4B**) we did indeed observe a slight increase in corpse number (statistically significant in **Fig 4B**, insignificant in **Fig. 4C**, see **suppl. Tab s1**). However, restoring *mpk-1a* expression in the *mpk-1(ga111ts)* background either in the entire soma or specifically in the sheath cells resulted in a clear reduction in corpse numbers, analogous to the germline-specific *mpk-1* RNAi experiment in **Fig. 4B**. Hence, the results of two independent experimental approaches presented in revised **Fig 4B** and **Fig. 4C** both indicate that the somatic *mpk-1a* isoform negatively regulates corpse formation, most likely because RAS/MAPK signaling is needed for apoptotic corpse clearing in the sheath cells. By contrast, the *mpk-1b* isoform in the germ cells promotes corpse formation. These data are also in line with our recent findings showing that LET-23 EGFR signaling in the sheath cells is necessary for corpse clearing (see Intl. Worm Meeting 2023 abstracts). We have rephrased this critical section of the manuscript (**lines 338f and 397f**) and clarified our conclusions (**lines 417f**).

As it is, the manuscript over-relies on using a weak gain-of-function let-60/ras mutation. The problem is that one might imagine the possibility that stronger (or weaker) gain-of-function mutations might produce different outcomes.

---

4. Hyperactivation of the RAS/MAPK pathway by *gap-1* and *gap-3(lf)* mutations also enhances germ cell apoptosis

As another way to hyperactivate RAS/MAPK signaling, we have used different combinations of loss-of-function mutations in the *gap-1* and *gap-3* genes that encode GTPase activating proteins (Stetak et al 2008) to achieve different levels of RAS/MAPK hyper-activation (**suppl Fig. S4F** and **lines 354**). As mentioned in the **legend to suppl. Fig. S4**, we did not quantify homozygous *gap-3(lf); gap-1(lf)* double mutants because of their severely perturbed gonad morphology (similar to *let-60(ga89ts)* mutants grown for 24 hours or longer at 25°C). Though, also the homozygous double mutant animals contained large numbers of corpses. Interestingly, *gap-1(lf)* did not have an effect by itself, but it enhanced the heterozygous *gap-3(lf)* mutation.

Major Points

1. More convincing evidence is needed to support the idea that apical constriction functions in the induction phase rather than the execution phase.

As mentioned above under point 1, the tracking of mitochondrial loss indicates that constriction (**Fig. 1K**) and mitochondrial loss (**Fig. 1O**) occur simultaneously, at the onset of apoptosis.

2. The manuscript should more completely address and resolve the issue of the role of mpk-1 in germline apoptosis.

As described in detail above under point 3, we have used tissue specific RNAi and tissue-specific rescue experiments to distinguish between the different effects of the somatic *mpk-1a* and germline *mpk-1b* isoforms.

3. In their Abstract, the authors make the claim that "Through this mechanism, animals can adapt the rate of germ cell death and differentiation to changing environmental conditions." I could not find data in the manuscript supporting this claim.

This idea is based on Lopez et al (ref. 29) which showed that the food abundance sensed by DAF-2 regulates MAPK activity in the germline. We have removed this speculative argument from the abstract and now postulate that actomyosin-mediated constriction, the mechanism we describe in this manuscript, regulates the balance between germ cell death and oogenesis.

4. Lines 83-84. This statement furthers a misperception in the field. As discussed above, it may be that mpk-1 mutant female germ cells do not progress to the stage in which they undergo programmed cell death. In fact, there is evidence that MPK-1 might inhibit apoptosis.

As described in detail above under point 3, our data indicate that the apparent inhibition of apoptosis by MPK-1 stems from its somatic function in corpse clearing. As in the other references cited, we have used a brief inactivation of the conditional *ga111* allele in all experiments shown in **Fig. 4B** to exclude this effect, except for the data on the constitutive *mpk-1b(zh164)* allele.

5. The authors might consider analyzing a partial loss-of-function ced-3 allele, ced-3(n2427), to determine whether they might be able to enhance or suppress its effects by modulating the pathways they describe.

Since *ced-3(n717)* double mutants completely suppressed the effects of *ect-2* or *let-60* hyperactivation on apoptosis, it seems likely that weaker *ced-3* alleles will have a similar albeit weaker effect. We cannot see any additional information that could be gained from these experiments.

6. The authors analyze ect-2(gf); cep-1(lf) mutants and ect-2(gf); pch-2(lf) mutants. Given that perturbations of the actin cytoskeleton are pleiotropic, both pathways might be engaged. Thus, the analysis should include ect-2(gf) pch-2(lf); cep-1(lf) triple mutants.

Since neither of the double mutants showed a clear effect, we think a triple mutant will not give any additional information.

7. Lines 328-329 and subsequent text. The manuscript inadequately addresses the possibility that mpk-1a functions in sheath cells to regulate engulfment. Although

the generation of a germline-specific mpk-1 allele is useful, it doesn't resolve the possibility that the absence of apoptotic germ cells when mpk-1 activity is depleted from the germline is a secondary consequence of the meiotic progression defect. The authors might also examine ect-2(gf); mpk-1(ga111ts) mutants in addition to mpk-1(ga111ts) single mutants.

ect-2(zh8); mpk-1(ga111) double mutants were examined in **Fig 6A**.

As discussed in detail above under point 3, we now show that sheath cell-specific expression of *mpk-1a* suppresses the slightly enhanced apoptosis observed in *ga111* mutants at the restrictive temperature and reduces corpse numbers below wild-type levels (**Fig. 4C**). Conversely, soma-specific *mpk-1* RNAi significantly enhanced corpse numbers (**Fig. 4B**), which provides further evidence for the inhibitory effect of MPK-1A in the sheath cells, likely due to a function in corpse clearance downstream of LET-23 EGFR.

8. Lines 574-576. The authors state, "Here, we show that pachytene stage germ cells smaller than their neighbors are selectively eliminated through apoptosis, probably to make space for and donate their resources to the surviving germ cells entering oogenesis." If, as prior work indicates, germ cell shrinkage is a key element of the execution phase (see Raiders et al., 2018), the reason the smaller germ cells are eliminated is that they are smaller because they have already adopted the apoptotic fate and begun to shrink. If so, the expectation would be that the smaller germ cells had already undertaken the first known step of the apoptotic pathway in which mitochondria exit from the germ cells into the rachis prior to shrinking.

As discussed above under point 1, our new mitochondria tracking experiments (**Fig. 1L-O**) indicate that germ cell shrinkage occurs at the onset of apoptosis simultaneously with the loss of mitochondria.

Minor Points

1. Line 40-41. The apoptotic germ cells have already adopted a female fate. So, it would be preferable to state something like, "before they differentiate as oocytes."

corrected (see also reviewer 1)

2. Lines 44-46. Physiological apoptosis also removes binucleate germ cells (Raiders SA, Eastwood MD, Bacher M, Priess JR. Binucleate germ cells in Caenorhabditis elegans are removed by physiological apoptosis. PLoS Genet. 2018 Jul 19;14(7):e1007417. doi: 10.1371/journal.pgen.1007417. PMID: 30024879; PMCID: PMC6053125).

As described above under point 2, we have counted binucleate cells and found no increase in *ect-2* or *let-60 gf* mutants.

3. Lines 46-49. This statement should be modified because egl-1 is dispensable for physiological germ cell apoptosis.

corrected

4. Line 51. 1,000.

corrected (see also reviewer 1)

5. Lines 79-80. Sustained MPK-1 activation in pachytene requires the presence of sperm (Lee MH, Ohmachi M, Arur S, Nayak S, Francis R, Church D, Lambie E, Schedl T. Multiple functions and dynamic activation of MPK-1 extracellular signal-regulated kinase signaling in Caenorhabditis elegans germline development. Genetics. 2007

Dec;177(4):2039-62. doi: 10.1534/genetics.107.081356. PMID: 18073423; PMCID: PMC2219468.)

included reference
